# A Ubiquitously Conserved Cyanobacterial Protein Phosphatase Essential for High Light Tolerance in a Fast-Growing Cyanobacterium

**DOI:** 10.1128/spectrum.01008-22

**Published:** 2022-06-21

**Authors:** Patricia L. Walker, Himadri B. Pakrasi

**Affiliations:** a Department of Biology, Washington University, St. Louis, Missouri, USA; Oklahoma State University

**Keywords:** cyanobacteria, high light, stress tolerance, serine/threonine phosphatases

## Abstract

Synechococcus elongatus UTEX 2973, the fastest-growing cyanobacterial strain known, optimally grows under extreme high light (HL) intensities of 1,500–2,500 μmol photons m^−2^ s^−1^, which is lethal to most other photosynthetic microbes. We leveraged the few genetic differences between *Synechococcus* 2973 and the HL sensitive strain Synechococcus elongatus PCC 7942 to unravel factors essential for the high light tolerance. We identified a novel protein in *Synechococcus* 2973 that we have termed HltA for High light tolerance protein A. Using bioinformatic tools, we determined that HltA contains a functional PP2C-type protein phosphatase domain. Phylogenetic analysis showed that the PP2C domain belongs to the bacterial-specific Group II family and is closely related to the environmental stress response phosphatase RsbU. Additionally, we showed that unlike any previously described phosphatases, HltA contains a single N-terminal regulatory GAF domain. We found *hltA* to be ubiquitous throughout cyanobacteria, indicative of its potentially important role in the photosynthetic lifestyle of these oxygenic phototrophs. Mutations in the *hltA* gene resulted in severe defects specific to high light growth. These results provide evidence that *hltA* is a key factor in the tolerance of *Synechococcus* 2973 to high light and will open new insights into the mechanisms of cyanobacterial light stress response.

**IMPORTANCE** Cyanobacteria are a diverse group of photosynthetic prokaryotes. The cyanobacterium *Synechococcus* 2973 is a high light tolerant strain with industrial promise due to its fast growth under high light conditions and the availability of genetic modification tools. Currently, little is known about the high light tolerance mechanisms of *Synechococcus* 2973, and there are many unknowns overall regarding high light tolerance of cyanobacteria. In this study, a comparative genomic analysis of *Synechococcus* 2973 identified a single nucleotide polymorphism in a locus encoding a serine phosphatase as a key factor for high light tolerance. This novel GAF-containing phosphatase was found to be the sole Group II metal-dependent protein phosphatase that is evolutionarily conserved throughout cyanobacteria. These results shed new light on the light response mechanisms of *Synechococcus* 2973, improving our understanding of environmental stress response. Additionally, this work will help facilitate the development of *Synechococcus* 2973 as an industrially useful organism.

## INTRODUCTION

Photosynthesis requires light energy, but all photosynthetic organisms have a limited capacity for light utilization. As a result, excess absorbed light causes increased production of reactive oxygen species (ROS) within the photosynthetic machinery, leading to severe damage to photosystem II (PSII) (1). The accumulation of photodamage reduces photosynthetic capacity, referred to as photoinhibition. Maximum photosynthetic efficiency is achieved when all light energy absorbed is utilized for carbon assimilation. Under this optimal condition, the rate of PSII damage is lower than the rate of PSII repair and photoinhibition does not occur (1, 2). Because avoiding prolonged photoinhibition is vital to survival, organisms have evolved numerous mechanisms to detect changes in light intensity, prevent photodamage, and repair damage to PSII (3). Although the photosynthetic reaction centers are identical throughout oxygenic photosynthetic organisms, many cyanobacteria have developed adaptation strategies that allow them to survive under high light (HL) conditions (1, 2, 4).

An important mechanism to prevent excess absorbed energy from causing photoinhibition is to reduce light absorption by decreasing the amount of chlorophyll and phycobilisome (PBS) and by lowering the PSI/PSII stoichiometry (5, 6). Another initial response to prevent photodamage and acclimate to increased light is by removing excess energy through quenching. Nonphotochemical quenching (NPQ) can occur through the orange carotenoid protein (OCP) as well as state transitions that redirect light energy between PSI and PSII (3). Quenching can also involve high-light-inducible proteins (HLIPs) (6, 7). However, since photodamage to PSII occurs under oversaturating light, an essential method to overcome photoinhibition is to repair PSII (7). ROS are inadvertently produced from the energy transfer in PSII, and the accumulation of ROS prevents PSII repair (2, 8). To allow for PSII repair, cyanobacteria have evolved a variety of antioxidant molecules such as carotenoids to eliminate ROS (6).

Cyanobacteria have developed various regulatory mechanisms to respond to high-intensity light, with many of these acclimation mechanisms mediated at the transcriptional level. One aspect that is not well understood of the stress response pathways is how stress is detected. Some cyanobacteria sense light through GAF (for cGMP-specific phosphodiesterase, adenylyl cyclase, and FhlA) domain-containing photoreceptors that bind bilin pigments (9). The enrichment of these photoreceptors is critical for acclimation to HL through mediation of signaling pathways that regulate HLIP, sigma factors, and pigments (5, 7, 10). Having a variety of signal transduction networks allows for detection and response to environmental changes. Members of the Mn^2+^/Mg^2+^-dependent protein phosphatase family known as PPM or PP2C provide rapid and reversible posttranslational modifications that play important roles in environmental response (11). In Gram-positive bacteria, environmental stress activates the stressosome complex that leads to the activation of the PP2C family phosphatase RsbU, a regulator of sigma factor SigB, to bring about stress response that enhances survival (12, 13). Further, regulation of phosphorylation levels of the cyanobacterial photosynthetic apparatus and metabolic enzymes was recently found to be pivotal for responding to environmental conditions (14). However, little is known about the identity and physiological role of phosphatases in cyanobacteria.

Many of the known HL response mechanisms are accompanied by decreased growth rates. This highlights the need to study the adaptive mechanisms evolved by HL tolerant species that grow optimally under HL conditions. Synechococcus elongatus UTEX 2973 (*Synechococcus* 2973) is one of the fastest-growing cyanobacteria, growing optimally under extreme HL up to 2,400 μmol photons m^−2^ s^−1^ (15, 16) with a high photosynthetic efficiency (17). Both *Synechococcus* 2973 and other HL tolerant species are found to upregulate PSI (16, 18) as well as electron carriers in response to HL, thus increasing photosynthetic capacity. *Synechococcus* 2973 shares over 99% genome similarity with the model strain Synechococcus elongatus PCC 7942 (*Synechococcus* 7942), yet the strains have distinct HL responses. Similar HL conditions result in fast growth of *Synechococcus* 2793 but decreased viability of *Synechococcus* 7942. Intriguingly, these strains do not encode an OCP, so mechanisms of NPQ remain unknown. The total genetic differences between these strains comprised 53 single nucleotide polymorphisms (SNPs), one large inversion, and a small deletion (15). We hypothesized that these genetic differences induce differential physiological responses that alter HL tolerance. Through the comparison of these two strains, the genetic markers for differential phenotypic traits can be identified. Using a CRISPR/Cas12a genome editing system, 36 mutants in Synechococcus 2973 containing substituted alleles from Synechococcus 7942 were constructed without antibiotic markers (19). Previous analysis of these SNPs uncovered key genes for fast growth and natural competency (20, 21).

In this work, we report on the finding of an uncharacterized protein, which we term HltA, that is vital for HL tolerance in *Synechococcus* 2973. Bioinformatic characterization highlighted a resemblance of the C-terminal domain with the PP2C family phosphatase domain of RsbU and showed that the PP2C domain retains the conserved residues and structure necessary for its function. We found that the N-terminal signaling domain does not have strong homologs and that this combination of domains is unique to HltA. We further showed that this protein is conserved throughout cyanobacteria. Moreover, inactivation of *hltA* led to impaired viability during HL growth. Thus, *hltA* has an essential role in cyanobacterial high light tolerance.

## RESULTS

### Identification of high light sensitive mutants.

**FIG 1:**
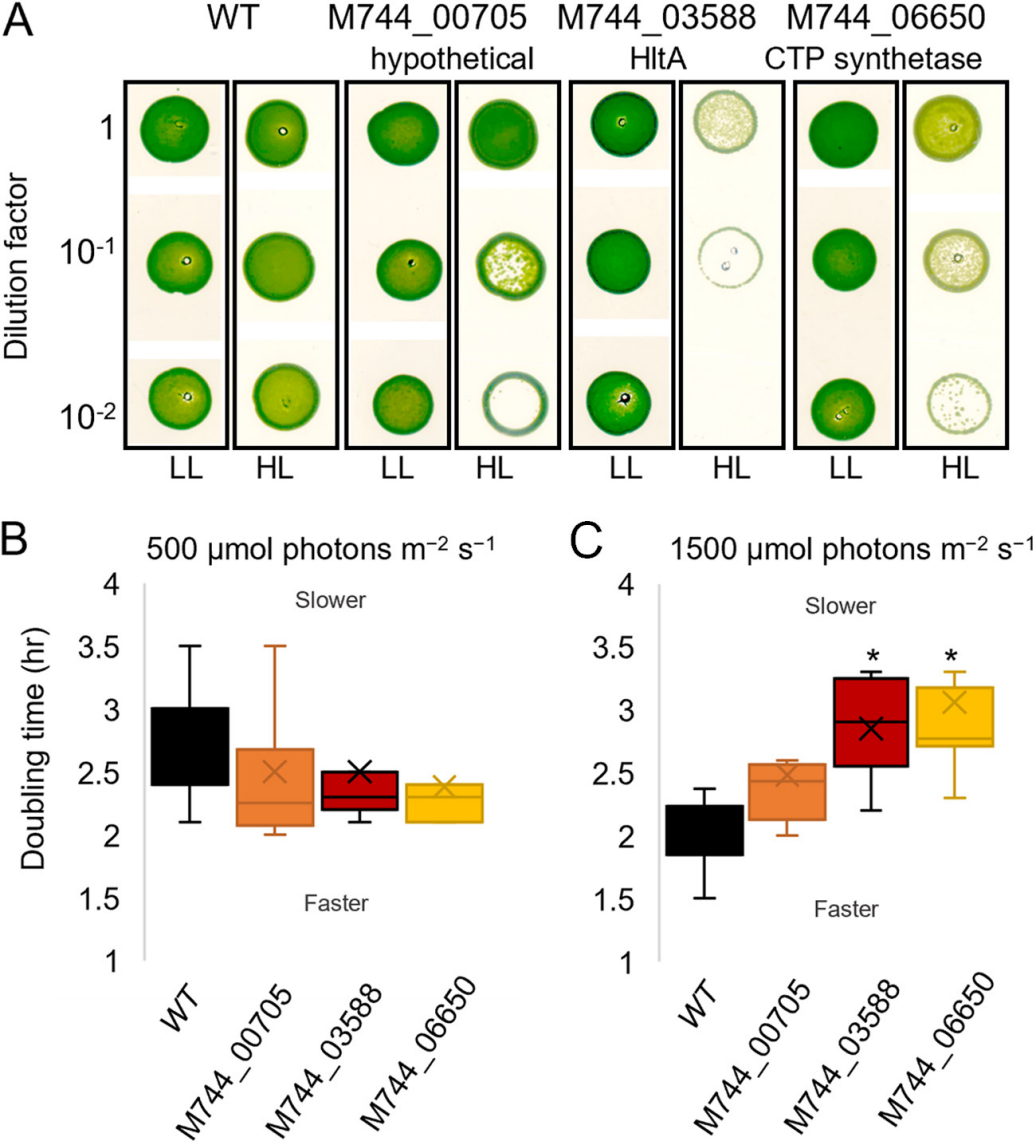
*Synechococcus* 2973 allele reversion mutants decrease high light tolerance. (A) High light sensitivity screening plate assay performed on wild-type and single allele reversion mutants of select loci. The representative plate of *n* ≥ 3 shown; HL_P_, 500 μmol photons m^−2^ s^−1^, and LL_P_, 50 μmol photons m^−2^ s^−1^. (B) Box plot representation of doubling times of wild-type *Synechococcus* 2973 and single SNP point mutants at ML_L_, 500 μmol photons m^−2^ s^−1^ and (C) HL_L_, 1500 μmol photons m^−2^ s^−1^. Mean doubling time calculated from at least four experiments. Asterisk indicates *P* value = 0.01–0.05, from Dunnett’s multiple-comparison test for each strain compared to *Synechococcus* 2973. Box plots indicate median (line), mean (*x*), and first and fourth quartile (whiskers) of doubling times.

**FIG 2:**
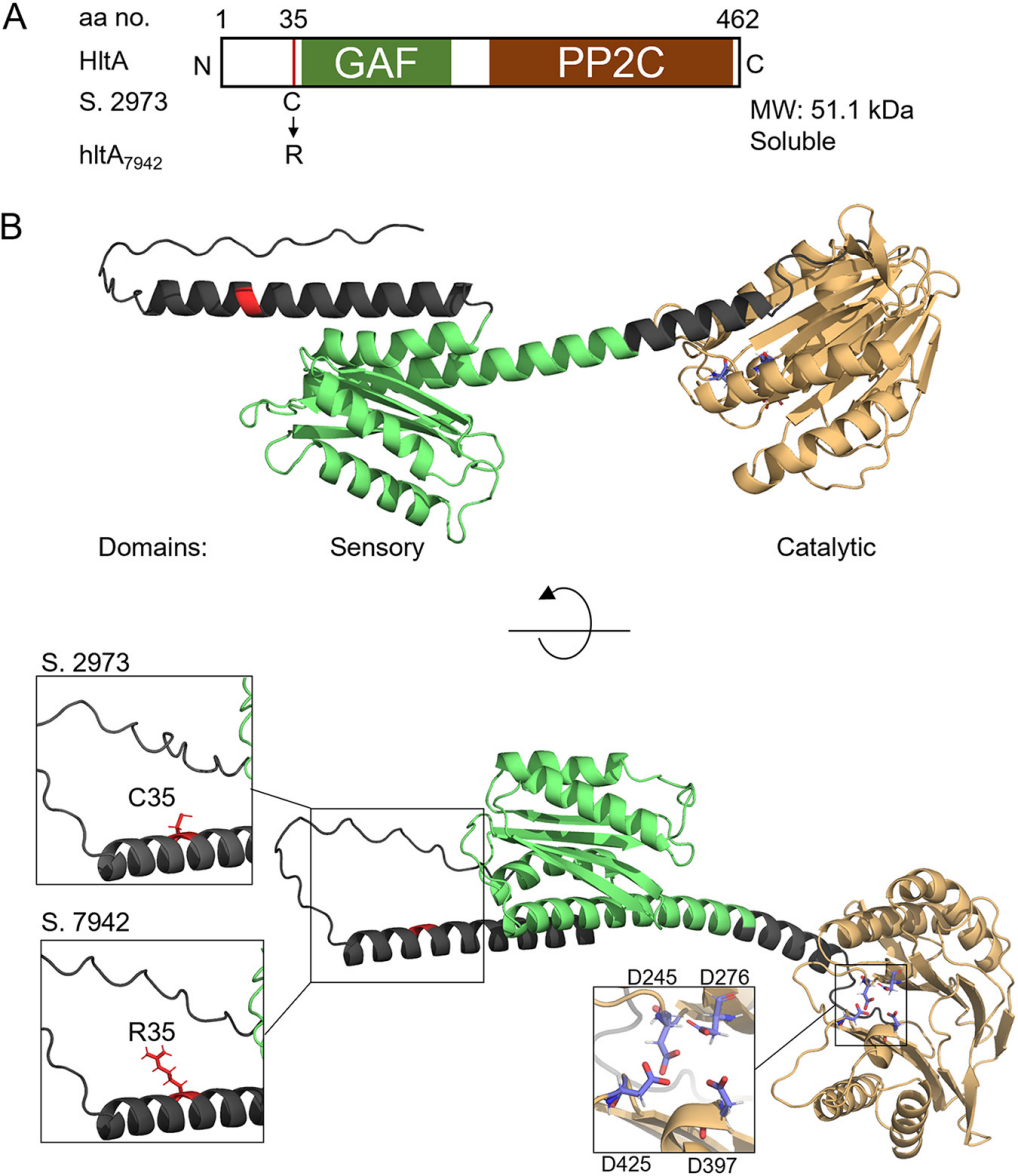
The modeled structure of the HltA protein. (A) Schematic diagram of the domain structure with SNP variation and characteristics shown below. (B) Ribbon diagram of the predicted structures of *Synechococcus* HltA protein, generated using AlphaFold (23). Location of the SNP shown in red, GAF domain shown in green, PP2C domain shown in light brown, active site Asp residues involved in metal binding shown in violet. Left insets highlight the Cys and Arg SNPs at residue 35; right inset shows the positioning of putative metal coordinating residues of the active site.

To elucidate alleles responsible for the differential light tolerance between *Synechococcus* 7942 and *Synechococcus* 2973, a series of previously generated *Synechococcus* 2973 CRISPR/Cas12 mutant lines (20) were assayed for their HL tolerance. These mutant lines contained *Synechococcus* 2973 alleles modified to the *Synechococcus* 7942 versions and a *Synechococcus* 2973 small plasmid deletion strain. In total, 36 mutant strains (Table S1) were assayed for a loss of HL tolerance through dilution series spot plates and verified for HL_L_ (1,500 μmol photons m^−2^ s^−1^) sensitivity in liquid growth assays. The dilution spot plate assays were more sensitive at detecting slight changes to HL tolerance as there was no shading of cells allowing HL_P_ exposure to remain consistent. Because of the shading and movement that occurred in liquid cultures, a higher light intensity was used to test HL tolerance when assaying liquid cultures. HL screening of all mutants led to the identification of three alleles of the most significant SNPs that individually decreased viability under HL. The corresponding genes encoded HltA, a hypothetical protein, and CTP synthetase (Fig. 1A), respectively. In liquid cultures, all three mutants grew at similar rates under medium light (ML_L_, 500 μmol photons m^−2^ s^−1^) conditions (Fig. 1B), suggesting that these mutations did not affect normal cell growth. In liquid culture, two of the three mutants grew significantly slower than the wild-type strain under HL (Fig. 1C). The SNP with the most significant effect was in the putative serine phosphatase locus M744_03855, which will be referred to as *hltA* (High light tolerance protein A). The *Synechococcus* 2973 *hltA* SNP mutant (*hltA*_7942_) contains an Arg, as in *Synechococcus* 7942, in place of the native Cys, as the 35th residue of the encoded protein (Fig. 2A).

### Sequence analysis of HltA.

M744_03588 has been annotated as a guanylate cyclase (NCBI: AJD57037.1) as well as a serine phosphatase (Uniprot: Q8GIR9) in different databases, as previously noted (15). To understand the nature of this protein, we conducted a series of analyses to examine its sequence features, dissect the domain components, and establish relationships with other well characterized proteins. The *hltA* gene is flanked by genes that are transcribed in the same direction, although in a previous analysis of the transcriptional start site, the gene did not appear to be in an operon (22). The HltA protein has a predicted molecular mass of 51.1 kDa with 462 residues. Among them, residues 227–461 form a PP2C-family SpoIIE domain [Pfam: PF07228]. Remarkably, in contrast to any previously characterized PP2C, HltA also contains a GAF domain near the N terminus (residues 59–200) (Fig. 2A and B). A BLASTP homology search of HltA revealed that it shared the highest degree of similarity (>50%) to sequences in other cyanobacteria. To search for homologs outside of the cyanobacterial clade, a BLASTP search excluding cyanobacteria was conducted that yielded 38 uncharacterized bacterial homologs, with no hits from eukaryotic organisms (Table S2). The closest characterized sequence is the PP2C-family phosphatase RsbU from Bacillus subtilis, which shares 27.8% sequence identity. However, RsbU does not have a GAF domain. We next modeled the structure using AlphaFold (23), which predicts protein structures with high accuracy even without known similar structures (Fig. 2B).

### HltA is a PP2C-family phosphatase.

**FIG 3:**
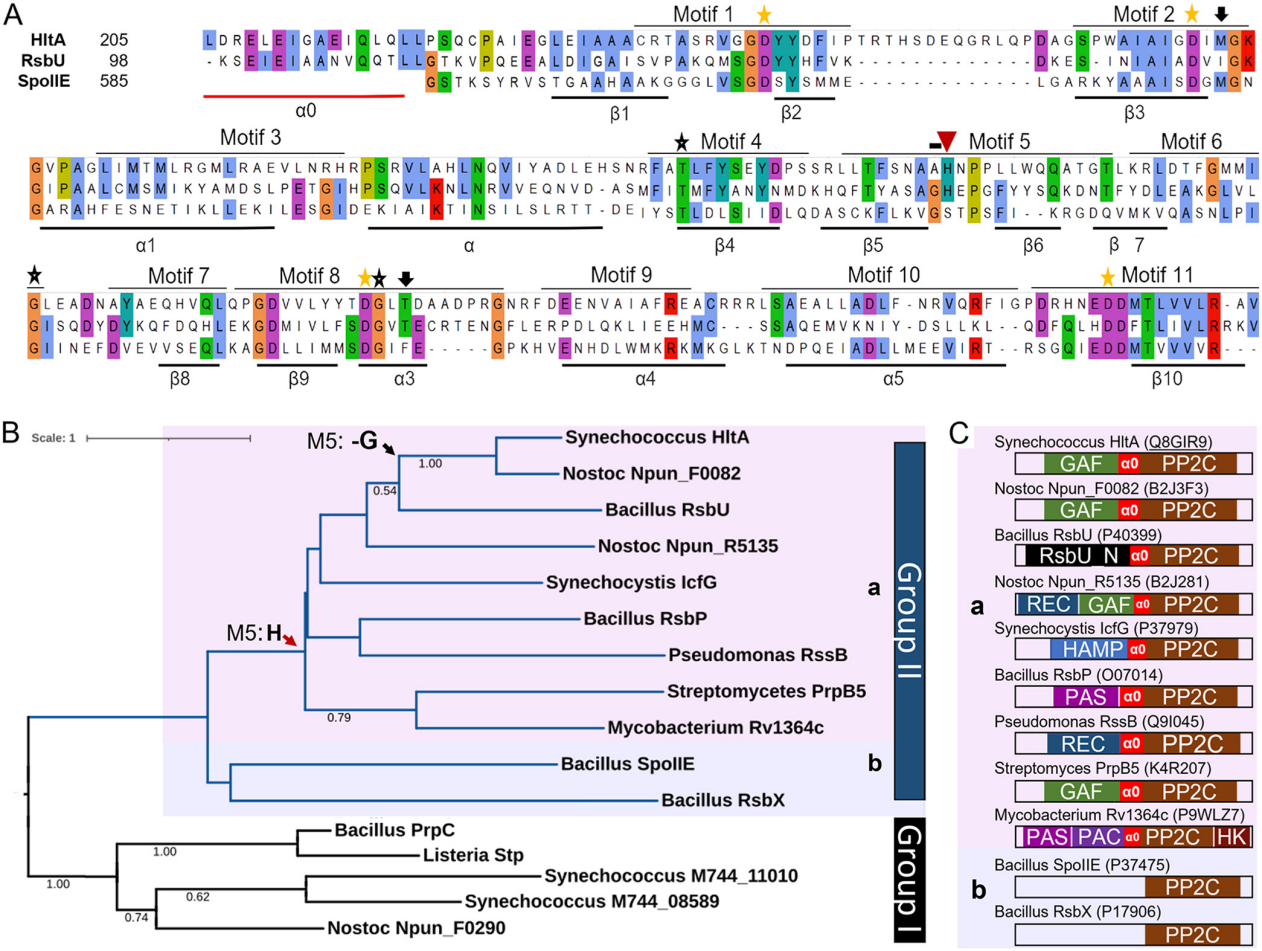
HltA is a Group IIa bacterial PP2C phosphatase. (A) Multiple sequence alignment of the 11 conserved PP2C motifs and preceding α0, of HltA from *Synechococcus* 2973, and RsbU and SpoIIE from B. subtilis. Stars indicate highly conserved residues across PP2Cs; yellow stars denote essential metal-binding residues. Black arrows indicate residues that may support catalytic activity; the red arrow indicates conserved residue in Group IIa sequences. Underlined sequences indicate predicted secondary structures of HltA, labeled according to structure of SpoIIE (27). Identical residues are highlighted. Secondary structures below the alignment represent properties from SpoIIE and are supported by PSIPRED structural predictions for HltA. (B) Maximum likelihood tree of *hltA* and bacterial PP2C family phosphatases. PP2C domain sequences were aligned with MUSCLE and used to infer the maximum likelihood protein tree. Branch color indicates PP2C group: pink highlight for Group IIa, blue highlight for Group IIb. The red arrow indicates conserved H in motif 5. The tree was generated using MEGA-X with LG+G+I model; supporting values from 1,000 bootstrap greater than 0.5 are shown on branches. The tree is drawn to scale, with branch lengths measured in the number of substitutions per site. (C) Schematic representation of domain architectures (SMART and Pfam domains) of Group II PP2Cs, with Uniprot ID. Protein domains shown are GAF (SM00065), PP2C (SM00331), HAMP (SM00304), HatPase Histidine Kinase (HK, SM000387), Receiver (Rec, SM00448), PAS (SM000091), and PAC (SM000086). α0 linker between sensory domains and PP2C domain identified using Alphafold. Domain architectures are not drawn to scale.

Members of the PP2C family are characterized by the presence of 11 signature motifs that correspond to the structure and function of the catalytic domain (24). HltA has all four metal-binding Asp residues (11, 25) (Fig. 2B) within the 11 conserved motifs (Fig. 3A). The number of bound metal ions is a differentiating characteristic of the Group I and Group II PP2Cs. Members of Group I such as tPphA bind three metal ions, whereas SpoIIE, a Group II member, binds two metal ions (26). We observed that HltA shares some important residues with tPphA, including a Met in motif 2 that supports the active site, and the second Asp in motif 8 involved in stabilizing metal binding (Fig. 3A). Notably, HltA belongs to Group II (Fig. 3B), and the absence of a Gly in motif 5 suggests that it, like other Group II members, cannot bind a third metal ion. The predicted structure of HltA displays a conserved composition of secondary structural elements composed of 10 β sheets and 5 α helices (Fig. 2B and Fig. 3A) observed in the active site of SpoIIE (27). The structure also revealed that HltA possesses an additional α helix (α0) preceding the PP2C core domain (Fig. 3A and C), a structural feature that may function as a regulatory module to control its phosphatase activity (28, 29).

To explore relationships between HltA and previously characterized proteins, we searched for structurally related proteins using JPRED4, which resulted in several hits in the PP2C family: RssB (PDB: 3f7a) (e-value 4e^−09^) and Rv1364c (PDB: 3ke6) (e-value 1e^−06^). We note that despite the significant degree of structural conservation, the level of sequence identity was low (~24.8%). To evaluate similarity of domains that may lead to functional inferences, PP2C domain sequences from *Synechococcus* 2973, *Synechocystis* 6803, Nostoc punctiforme, *Streptomyces* sp., and previously characterized PP2Cs were used to construct a phylogenetic tree. The maximum likelihood tree in Fig. 3B shows separation of the Group I eukaryotic-like PP2Cs that contain the motifs 5b and 5c and Group II bacterial-specific PP2Cs (11), which reflects the functional differences between these groups. Within Group II, we observed two subclades, one of which, Group IIa, contains a known conserved His residue (H348) within motif 5 (30), indicated by a red arrow in Fig. 3A and B. Additionally, we found that HltA and the HltA ortholog Npun_F0082 lack a Gly. The replacement of Gly with Ala (A347) in motif 5 was observed in all cyanobacterial HltA orthologs. As seen in the phylogenetic analysis, the closest characterized phosphatase to the PP2C domain of HltA is RsbU (Fig. 3B). Synechococcus elongatus encodes three probable PP2C-type phosphatases, none of which have been previously characterized. The two other putative PP2C-type phosphatases belong to Group I and do not contain any sensory domain (Fig. 3B and C). Together, our analysis suggests that the HltA phosphatase domain likely functions similarly to RsbU and differs in functionality from the other PP2Cs in *Synechococcus* 2973.

### HltA is ubiquitous in cyanobacteria.

**FIG 4:**
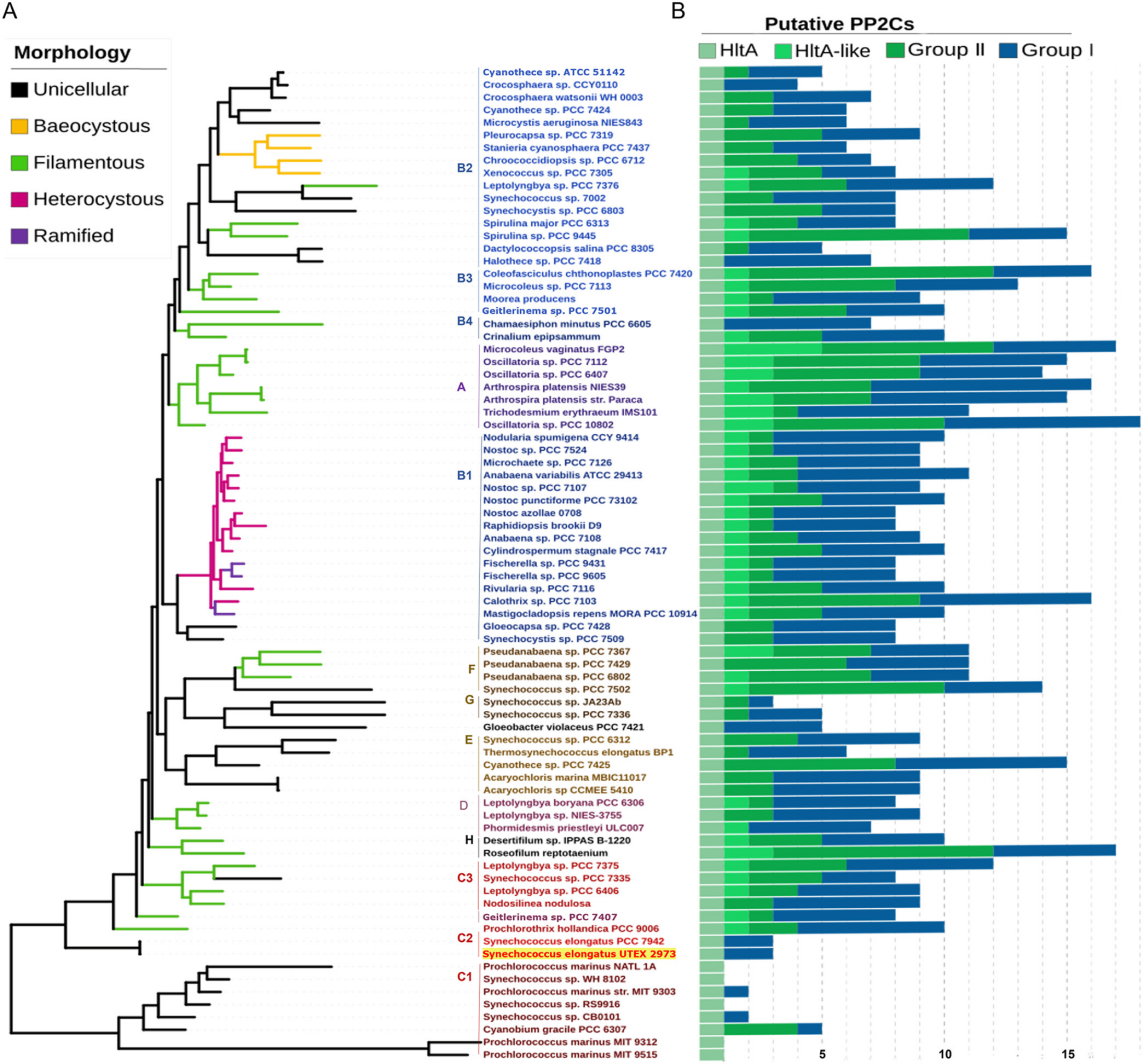
HltA is conserved throughout cyanobacteria. (A) Maximum likelihood phylogeny of *hltA* across cyanobacteria and distribution of additional HltA-like proteins. Branches are color-coded according to the morphological trait. Taxa names are colored by phylogenetic species clades (31). (B) The number of putative PP2C genes present in each genome is divided into four categories. Group II PP2Cs in green, with HltA orthologs, HltA-like sequences (which include PP2C sequences with at least one GAF domain) subcategories, and Group I PP2Cs in blue. Sequence homology search was carried out using the IMG database and aligned with MUSCLE with ICFG and RsbU outgroups (not shown). The tree was created using MEGAX software, bootstrapped 1,000 replicates. Outgroups and bootstrap values are shown in Fig. S3.

To evaluate the conservation and evolutionary relationship of HltA in cyanobacteria, we searched for *hltA* orthologs in 410 cyanobacterial genomes, available in the Integrated Microbial Genome database (IMG: https://img.jgi.doe.gov). We selected orthologs with sequence similarity spanning both domains, >50% identity, with a 0.01 e-value cut off. We identified orthologs in 400 genomes, including the nonphotosynthetic UCYN-A and the most primitive cyanobacterium *Gloeobacter violaceous* PCC 7421 (Table S4). The ortholog sequences from the remaining 10 genomes mostly comprised *Prochlorococcus* and *Synechococcus*. All genomes contained a single copy of the *hltA* ortholog. In two *Arthrospira* strains, *hltA* appeared to be misannotated as two separate genes, separating the GAF and PP2C domains with only one start and one stop codon between the two. We selected 75 ortholog sequences from morphological and ecologically diverse cyanobacterial strains for phylogenetic analysis to investigate the relationship of *hltA* orthologs across cyanobacteria. The distribution of the resulting *hltA* tree topology maintained a high degree of similarity with the core cyanobacterial phylogeny (31–33) shown by the preservation of distinct clades A–H (Fig. 4A and Fig. S3), with little divergence in clade distribution. The most notable topological difference is of clade C (Fig. 4B), with the presence of subclades c1 and c2 at the bottom of the tree suggesting that these sequences are the most divergent ones. Clade C is predominantly composed of *Prochlorococcus* and *Synechococcus*, which are the fastest evolving lineages and have undergone significant genome reductions (32, 33). Additionally, a single species, *Geitlerinema* sp. PCC 7407, did not group together with its proposed clade (clade D).

We have hypothesized that the aberrant *hltA* clades could be attributed to Group II PP2C duplication events that could allow for sequence variability to occur. To assess this, we identified the number of putative PP2Cs in each genome and categorized these sequences based on Group I or Group II features, and more specifically Group II sequences encoding one or more GAF domains. In this analysis, we observed that HltA is the only PP2C-family phosphatase with orthologs in all cyanobacteria, shown by the light green *hltA* bar in all genomes (Fig. 4B). Interestingly, the possible Group II duplication events did not coincide with the aberrant clades, but rather with cellular morphologies. Unicellular species in subclades c1 and c2 predominantly encode a single copy of a Group II PP2C, the HltA ortholog. In contrast, subclades c3 and D contain filamentous species that encode multiple PP2C proteins with several copies of PP2Cs containing GAF domains (Fig. 4B). Filamentous and heterocystous cyanobacteria have a greater number of Group II PP2C genes, due to genome expansion events (33) and sensory domain recruitment to PP2Cs that possibly assisted in adaptation to different environments (11). Rather, the divergent *hltA* clades associated closer with genome complexity. Clades c1, b2, and A and *Geitlerinema* sp. PCC 7407 of clade D all have higher genome complexity, measured by sequence compositional complexity, which resulted from trends toward driven progressive evolution (33). The overall conservation of clades and presence of *hltA* throughout cyanobacteria suggest that its function is necessary and was established early in cyanobacterial evolution.

### HltA is essential for high light tolerance of *Synechococcus* 2973.

**FIG 5:**
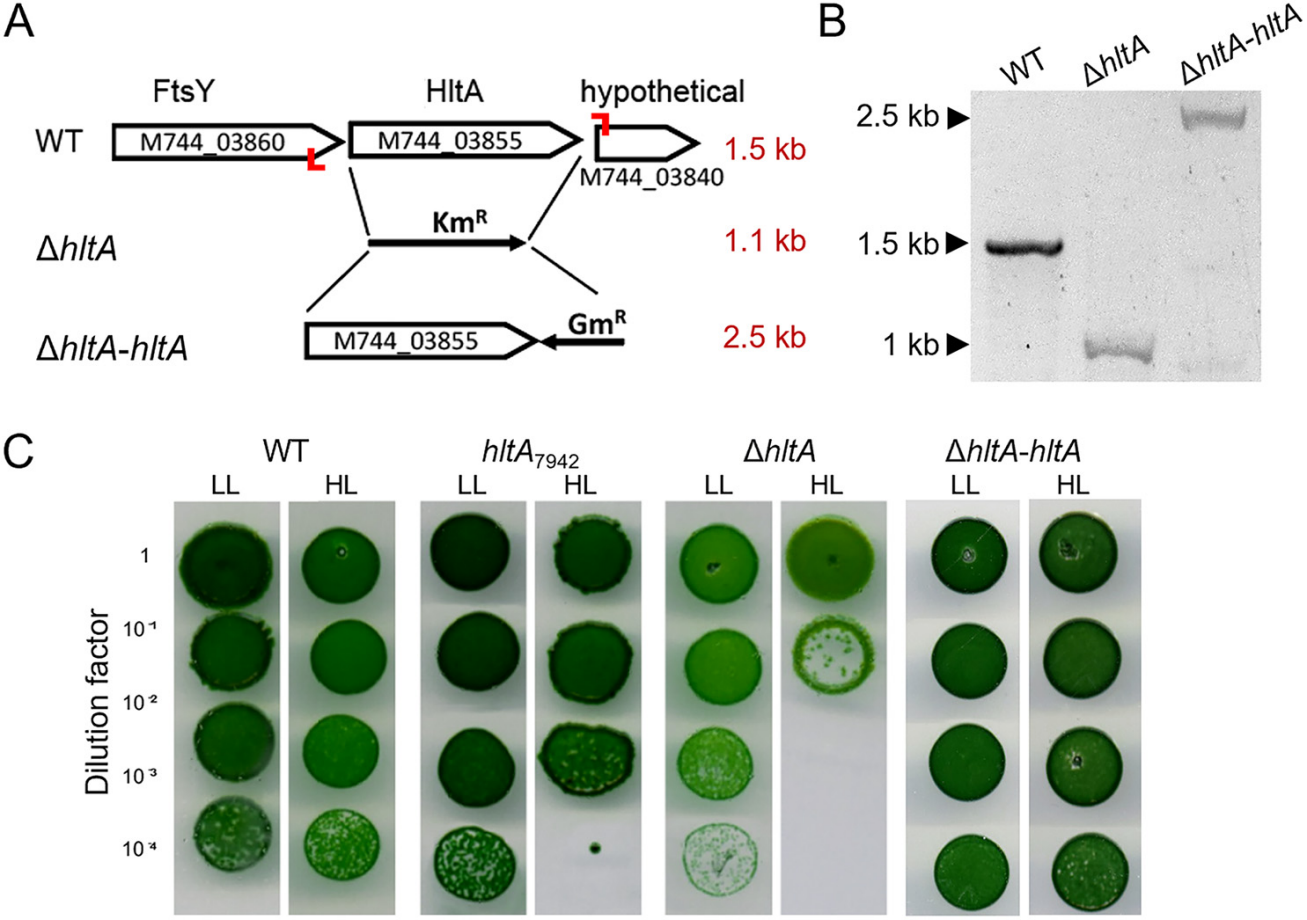
Mutations to *hltA* cause sensitivity to high light in *Synechococcus* 2973. (A) Organization of *hltA* and flanking DNA region containing M744_03860 (FtsY), M744_03855 (HltA), and M744_03840 (hypothetical protein), the position of the inserted kanamycin resistance cassette (KmR) to generate the Δ*hltA* strain, and replacement of KmR with *hltA* and gentamicin resistance cassette (GmR) to generate the complementation strain Δ*hltA*-*hltA*. Red bars indicate the location of primers used in panel B; red text indicates the size of the PCR product. (B) Deletion and complementation of *hltA* in the Δ*hltA* and Δ*hltA-hltA* strains were tested by PCR. (C) Growth of WT and *hltA* mutants from HL dilution spot plate assays. Strains grown on plates with their respective antibiotic resistance. HL_P_ (500 μmol photons m^−2^ s^−1^), LL_P_ (50 μmol photons m^−2^ s^−1^).

To verify that HltA is important for HL growth, we engineered a *hltA* deletion strain by replacing the full-length *hltA* with a Kan^R^ cassette (Fig. 5A). Since *Synechococcus* 2973 maintains multiple genome copies, the replacement of *hltA* was checked with PCR primers set outside the homology arms (Table S3) and growth on antibiotic plates. The *hltA* mutant obtained after transformation was partially segregated, and the wild-type gene could still be detected by PCR. Repeated subculturing under higher antibiotic concentrations was performed, resulting in the *hltA* deletion mutation (Fig. 5B). This deleted *hltA* mutant strain (Δ*hltA*) exhibited HL sensitivity more severe than the *hltA*_7942_ mutant (Fig. 5C). To confirm that the sensitivity to HL was caused by the *hltA* mutation, we introduced the full-length *hltA* gene back into the Δ*hltA* strain (Fig. 5A) and examined the complemented strain for the restoration of HL_P_ tolerance. As shown in Fig. 5C, the complemented strain has regained its HL tolerance, pointing to the critical nature of this gene for HL adaptation.

To further understand the mechanisms of HltA, we assessed photosynthesis parameters of the mutant in response to HL treatment. Wild-type and *hltA* mutant cultures subjected to ML_L_ and HL_L_ treatments were monitored for differences in pigment levels recorded by the absorption spectra, and for changes in energy transfer between PBS and photosystems. The absorbance and fluorescence emission spectra exhibited no significant difference between the mutants and wild-type strains (Fig. S1A-C). Additionally, quantum yield efficiency of PSII showed no difference between these strains in either light condition (Fig. S1D). These results indicate that HltA is not directly involved in regulating photosynthetic capacity.

### HltA is specific to high light stress response.

**FIG 6:**
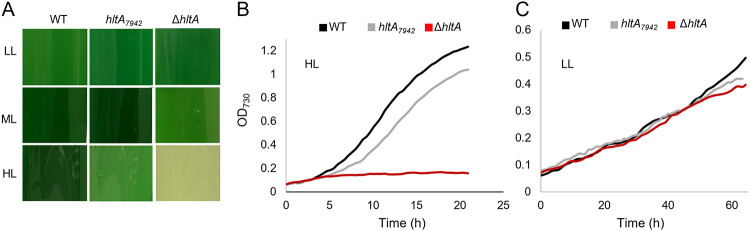
*hltA* mutant sensitivity is high light specific. (A) Phenotype of wild-type and *hltA* strains, grown at 42°C under different light conditions for 24 h. Cultures were grown with 1% CO_2_. (B) Growth curve of wild-type and mutant strains grown under HL_L_ (1,500 μmol photons m^−2^ s^−1^) and 42°C. (C) Growth of wild-type and mutant strains under LL_L_ (50 μmol photons m^−2^ s^−1^) and 42°C. Data shown represents three replicates.

To determine whether HltA affects more general stress pathways, we challenged the wild type and *hltA* mutants with various stress conditions. The mutants did not exhibit growth defects compared to the wild type under various nutrient-deprived or high salinity conditions (Fig. S2). All studies were performed at 38°C for of the comparison with *Synechococcus* 7942, which grows best at this temperature, although the optimum fast-growth temperature for *Synechococcus* 2973 is 42°C (16). Thus, we examined growth at 42°C under HL_L_, ML_L_, and LL_L_ (50 μmol photons m^−2^ s^−1^) conditions. Under HL_L_, both mutants exhibited acute growth defects, with the Δ*hltA* strain losing viability, while under ML_L_ only the Δ*hltA* mutant exhibited minor sensitivity (Fig. 6A and B). Since photoinhibition still occurs at the ML_L_ condition, growth at 42°C under a LL_L_ condition was necessary to isolate the effects of the increased temperature. Under LL_L_, we observed no differences in growth between the *hltA*_7942_ and wild-type strains, whereas the Δ*hltA* strain did not exhibit growth defects for the first 2 days of treatment (Fig. 6A and C). Since high temperature amplified the detrimental effects of the HL but was not harmful to the *hltA* mutants on their own, our analysis demonstrated that HltA is primarily involved in alleviating high light stress.

## DISCUSSION

*Synechococcus* 2973 optimally grows at some of the highest light conditions recorded for photosynthetic organisms (20). Despite the few genetic differences between *Synechococcus* 2973 and the HL sensitive *Synechococcus* 7942, only a few SNPs had been investigated for links to phenotypic differences. We set out to identify polymorphic alleles in *Synechococcus* 2973 to discover unexplored mechanisms of HL tolerance. We identified *hltA* to be essential for HL growth in the fast-growing *Synechococcus* 2973, as demonstrated by decreased colony formation and cell viability upon mutations to *hltA* (Fig. 1 and 5). In addition to these severe growth defects, there were also slight defects observed under low light conditions (Fig. 5C and 6). Given our finding that HltA is present in all cyanobacteria, including HL sensitive species, it is evident that HltA is necessary for tolerance to light at all intensities. Further, this decrease in growth was light specific and was not observed in any other general stress conditions. Therefore, we suggest *hltA* may be a key component to light response mechanisms specific to cyanobacteria.

Cyanobacterial phosphatases have been widely left unstudied in contrast with their cognate protein kinases, despite the observation that most proteins detected under different light conditions in cyanobacteria are phosphorylated on Ser or Thr residues (11). The RsbU phosphatase regulates alternative sigma factor B activity, which controls over 150 stress response genes (34). The similarity between *hltA* and *rsbU* (Fig. 3) suggests that *hltA* may regulate sigma factor activity. While neither Synechococcus elongatus species encode homologs of the RsbU protein target RsbV, analysis of the *hltA* neighborhood did not reveal any target candidates. Homologs to the stressosome are not present in *Synechococcus*, and *hltA* does not share identity with the N-terminal RsbU N domain that detects input from the stressosome pathway. Therefore, it is likely that HltA is part of a distinct environmental response pathway with regulation through the GAF domain. GAF domains are extremely widespread, known to bind to small molecules like cyclic nucleotides or chromophores, and take part in signal transduction pathways (9, 35). GAF domains are used by phytochromes to sense light through bound bilin pigments (36, 37), and are involved in plastoquinone redox sensing that can signal photosynthetic imbalances (38). Additionally, it is evident that the GAF domain of HltA is important, as demonstrated from the widespread conservation of the GAF-PP2C fusion that we observed throughout cyanobacteria. Analysis of the signals that activate the HltA GAF domain would provide insights into the regulation of HltA activity.

**FIG 7:**
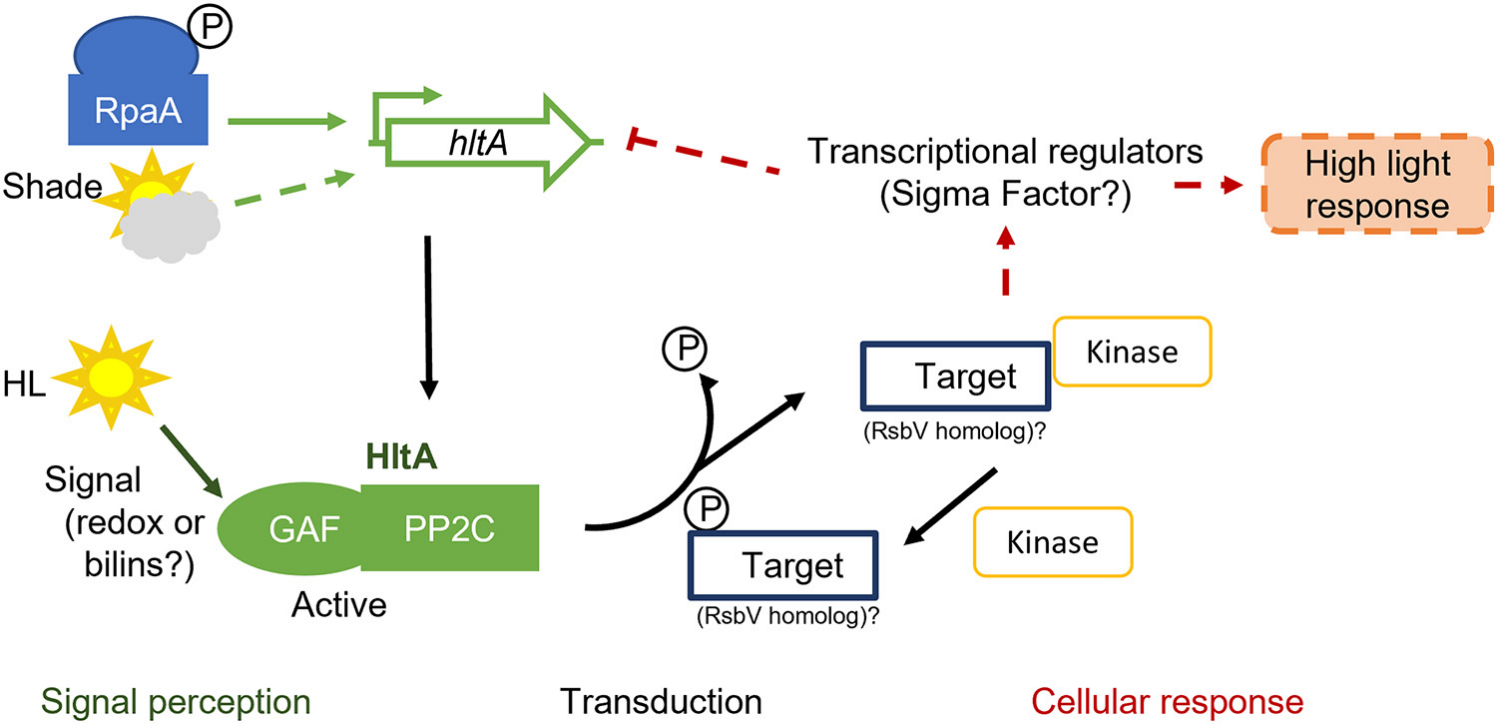
A model proposing regulation and function of HltA for *Synechococcus*. Phosphorylated RpaA controls *hltA* expression at dusk. Increases in light are detected by the GAF domain of HltA, which activates the HltA protein. Activated HltA dephosphorylates a target, possibly the RsbV homolog M744_05770 (NCBI: WP_011244281), which controls downstream regulation leading to high light response through unknown mechanisms, indirectly repressing expression of the *hltA* gene. An unknown kinase works in opposition to HltA, phosphorylating the HltA target. Phosphorylation events are indicated by “P”. Arrows indicate activation, T-bar represents inhibition. Solid and dashed lines indicate direct and indirect or unknown regulation, respectively.

*hltA* expression has been found to exhibit weak circadian oscillation (39) and was identified as a target of the master regulator of the circadian clock, RpaA (40). Therefore, it is plausible that *hltA* functions as a part of the circadian clock. To test circadian growth, cultures were entrained to a 12h light/12h dark cycle for three cycles, then exposed to constant light to observe the free running rhythm growth. We found that *hltA* mutations did not affect growth both during light/dark entrainment or following continuous light growth (Fig. S2). While *hltA* may not contribute to fitness under light–dark conditions, the transcriptional data do suggest its expression to be a function of both time-of-day and light intensity-dependent response (41). The homology to RsbU supports a role for HltA in a partner-switching system that regulates stress response gene expression through an alternative sigma factor. Given the various evidence, we present a speculative model (Fig. 7) in which RpaA controls *hltA* expression (40), with HltA then sensing changes to light conditions via its N-terminal GAF domain. The light-induced activation stimulates dephosphorylation of the target, possibly the putative sigma factor B antagonist M744_05770, which shares 28% identity with RsbV. The HltA target can then bind to and sequester the antisigma factor kinase, freeing the alternative sigma factor to promote transcription of light stress response genes. In addition, activated HltA may indirectly block its expression in a negative feedback loop. This is supported by data showing the *hltA* gene product increases under shade conditions but decreases following high light pulse (41), as HltA is no longer needed. The model suggested here outlines directions for future studies investigating the partners and mechanisms of HltA as well as its role in the circadian clock.

We show that HltA belongs to the Group IIa phosphatases, a group that contains few characterized proteins (Fig. 3). Most PP2Cs have not been characterized, and many mechanisms of regulation are unknown (25). The majority of crystalized bacterial PP2Cs, such as tPphA and MtPstP (26), belong to Group I and contain a third metal-binding site and lack regulatory domains. In contrast, many Group II members are regulated by a diverse set of N-terminal domains (25). A PP2C with a lone N-terminal GAF domain has yet to be characterized. Further functional characterization of *hltA* is a promising avenue for advancing our understanding of Group II protein phosphatases.

The observation that PP2Cs are not found in all bacteria, together with the presence of the eukaryotic-like Group I PP2Cs in bacteria, led to the hypothesis that the PP2C family arose in eukaryotes and was acquired by certain prokaryotes through horizontal gene transfer (HGT) (11). This notion was challenged through phylogenetic analysis that suggested that PP2Cs originated in bacteria before inheritance of PP2Cs by eukaryotes, followed by eukaryotic-like Group I PP2Cs moving back into bacteria via HGT (30) Our phylogenetic analysis suggests *hltA* was fixed in the cyanobacterial genome before cyanobacterial clade divergence and therefore preceded eukaryotic PP2Cs. This is supported by the finding that *hltA* is part of the core gene set in many cyanobacterial genomes (42, 43). Genes involved in adaptation to different light intensities are believed to have been fixed in the core genome before cyanobacterial clades diverged (44). Transcriptomic evidence from *Nodularia spumigena* shows *hltA* ortholog expression clustered with genes that responded differently to environmental variables and showed enrichment for functional categories: environmental adaptation and transcription (45). Together these observations suggest that HltA is a core component of an environmental adaptation process conserved in cyanobacteria.

Based on the observations in this study, we have shown that HltA is a novel factor that facilitates a light-stress response. The single SNP adaptation to this protein in *Synechococcus* 2973 allows this strain to grow optimally under HL intensity. This SNP proceeds the predicted start of the N-terminal GAF domain, which may have been co-opted as a light-recognition module early during the evolution of cyanobacteria. Our study shows that *hltA* is maintained throughout cyanobacteria and presents an opportunity for further exploration of its physiological role in light response. *Synechococcus* encodes a single putative RsbV homolog (M744_05770) and 10 serine protein kinases (7 of which are putative). Functional studies to elucidate the partners of HltA in *Synechococcus* will lead to a greater understanding of high light tolerance and posttranslational modification networks in cyanobacteria.

## MATERIALS AND METHODS

### Cell growth and treatment.

The wild-type Synechococcus elongatus UTEX 2973 and constructed mutant strains in this study (listed in Table S1) were grown as described in Ungerer et al. (20) unless otherwise indicated. Cultures were maintained on BG11 agar plates at 38°C, 150 μmol photons m^−2^ s^−1^. To determine the effect of high light stress conditions on gene expression, cells were grown to midlogarithmic growth phase (optical density at 730 nm of 0.6–0.8) in an MC-1000 multicultivator (Photon Systems Instruments, Czech Republic), and diluted with fresh medium to an OD 730 of ~0.3 before exposure to high light (1,500 μmol photons m^−2^ s^−1^) for 30 min, 1 h, and 2 h, when cells were harvested. Growth experiments were repeated at least three times to confirm the growth patterns. Doubling times were calculated by fitting an exponential curve to the logarithmic section of the growth data (typically OD_720_ of <0.3) and using the slope, *m*, as *K*′ (*y* = *ke^mx^*). Doubling times were then calculated as ln(2)/*K*′. Mean doubling times were compared to *Synechococcus* 2973 using a one-way ANOVA and Dunnett’s multiple-comparison test.

### Spot plate assays.

Cultures with a starting OD (730) of 0.3–0.35 were spotted on plates in 5 μL spots and grown for 4 days on BG11 plates, under low light (50 μmol photons m^−2^ s^−1^) or high light (500 μmol photons m^−2^ s^−1^) at 38°C with 0.6% CO_2_ in a Caron plant growth chamber (Caron Products & Services, Inc., Marietta, OH, USA).

### Structural prediction.

Three-dimensional protein structural predictions were derived using AlphaFold (23), accessed using AlphaFold Colab (https://colab.research.google.com/github/deepmind/alphafold/blob/main/notebooks/AlphaFold.ipynb). Predicted secondary structures were also checked using HHpred, which queries the Pfam, PDB, and SCOP databases. Structural visualization and alignments used the PyMOL Molecular Graphics System (Schrödinger, LLC).

### Sequence analysis of *hltA* and genomic context.

Cyanobacterial genomes with finished and permanent draft genomes were compiled in the IMG database. *hltA* ortholog sequences were identified from the genome cart using the full-length M744_03855 sequence as our query. One representative gene sequence containing homology to both domains (>70% coverage on query gene) was selected per genome (listed in Table S4). The obtained candidate *hltA* homologs from each genome were further inspected for Interpro and Pfam conserved domain architecture (GAF and PP2C). Additional BLAST searches were executed using the standard parameters on NCBI for any cyanobacterial genomes of interest not found on IMG. Search for noncyanobacterial homologs was performed with NCBI BLASTP search excluding cyanobacteria with an >70% query coverage cut off, to cover homology to both domains (shown in Table S4).

### Sequence alignment and phylogenetic analysis of HltA.

Genomes for phylogenetic analysis were selected from Will et al. (32), and *hltA* orthologs from these genomes were aligned by multiple sequence alignment using MUSCLE with default settings. The phylogenetic tree was constructed in MEGAX by maximum likelihood method and the LG model (46). The tree with the highest log-likelihood was selected. Initial trees for the heuristic search were obtained automatically by applying Neighbor-Join and BioNJ algorithms to a matrix of pairwise distances estimated using the JTT model and then selecting the topology with superior log-likelihood value. A discrete gamma distribution was used to model evolutionary rate differences among sites. The rate variation model allowed for some sites to be evolutionarily invariable. The tree was drawn to scale, with branch lengths measured in the number of substitutions per site. Evolutionary analyses were conducted in MEGAX (47). The final dendrogram was visualized on the Interactive Tree of Life (iTOL) (48). Conserved domains were identified using SMART (Simple Modular Architecture Research Tool).

To identify putative PP2C genes, UniProt (http://www.uniprot.org/) and JGI Genome Portal (http://genome.jgi.doe.gov/) were used to search against cyanobacterial genomes used in the phylogenetic analysis. Putative PP2C gene counts were searched individually using a JGI pfam search, using keywords protein phosphatase 2C-like and SpoIIE, and validated through searches on UniProt.

### Construction of *hltA* deletion and complementation strains.

For the deletion of the *hltA* gene, *KanR* from PVZ321 and PUC backbone from PAM3103 (49) were amplified for Gibson assembly using primers (Table S2). Upstream and downstream genes of *hltA* were amplified from genomic DNA using primers Fragment 2.FOR and REV, and Fragment 4.FOR and REV. Gibson was assembled with fragments pUC and kanR to construct the depletion strain (Table S2).

For the complementation of the *hltA* gene, the entire coding region of M744_03855 was amplified by PCR using the *hltA* forward and reverse primers (Table S2). UP, PUC backbone, and DS were amplified from the deletion plasmid (primers HltA_DS and HltA_US) and assembled with the full-length *hltA* and gentamicin resistance cassette.

The resulting exiting vector plasmids were checked by sequencing and used to conjugate into *Synechococcus* 2973, and ex-conjugants were selected with 40 μg/mL of kanamycin or 20 μg/mL gent. The transformants were obtained and passed several times on BG11 plates supplemented with their appropriate antibiotic (gent concentration of 5–10 μg/mL, kanamycin 10–20 μg/mL) to achieve segregation. Mutants were verified by amplifying a region of chromosomal DNA with primers located outside of the homology region of the editing plasmids. Confirmed depletion mutants were used to conjugate with the complementation plasmid and selected for with gentamicin. All the primers used for cloning and plasmid construction are listed in Table S3.

### Functional validation of wild-type and *hltA* mutants under various stress conditions.

Liquid culture assays were carried out to ascertain the function of the mutated *hltA* and wild-type gene under various abiotic stress treatments (salt, nutrient, heat). Wild-type and mutant *Synechococcus* 2973 were grown for 4 days in BG11 media. Cultures were diluted and incubated at 38°C for up to 4 days under various abiotic stress conditions (high salt, and nutrient deprivation). For 42°C growth, cultures were diluted and allowed to adapt to Multicultivator conditions to an absorbance of (OD720) 0.5–0.6, then diluted once again to (OD720) 0.1 before incubating at 42°C with bubbling CO_2_ and various light intensities. Photos were taken after treatment. Stress tolerance was determined with respect to control cultures.

### Room temperature fluorescence kinetics.

The fluorescence parameter *F_v_*/*F_m_* (maximum quantum yield of PSII) was calculated as the equation of Fv/Fm = (Fm–Fo)/Fm, where Fo is the minimum fluorescence, Fv is the variable fluorescence, and Fm is the maximum fluorescence. Cultures were dark adapted for 3 min at room temperature before measured using a double-modulation fluorescence fluorometer, FL-200 (Photon Systems Instruments, Brno, Czech Republic). The instrument contained red LEDs for both actinic (20-μs) and measuring (2.5-μs) flashes and was used in the time range of 100 μs to 10 s.

### Fluorescence and absorption spectroscopy.

The fluorescence emission spectra of phycobilins and chlorophyll from whole cells of each strain were measured at 77 K on a Fluoromax-2 fluorometer (Jobin Yvon, Longjumeau, France). Excitation occurred at 580 nm and 435 nm respectively, and fluorescence emission was recorded between 600 nm and 750 nm and normalized to the readings at 730 nm. Whole-cell absorbance was measured on an Olis DW-2000 spectrophotometer, and data were analyzed with Olis Globalworks software (On-Line Instrument Systems, Bogart, GA, USA). All spectra were normalized at 730 nm to correct for differences in light scattering.
