# Supplementary material for: A Ubiquitously Conserved Cyanobacterial Protein Phosphatase Essential for High Light Tolerance in a Fast-Growing Cyanobacterium
Source: Microbiol Spectr. 2022 Jun 21;10(4):e01008-22. doi: 10.1128/spectrum.01008-22 (PMC9430166; doi:10.1128/spectrum.01008-22)
Supplement: Supplemental file 1 — Supplemental material. Download spectrum.01008-22-s0001.pdf, PDF file, 0.9 MB [file spectrum.01008-22-s0001.pdf]

Supplementary figures:

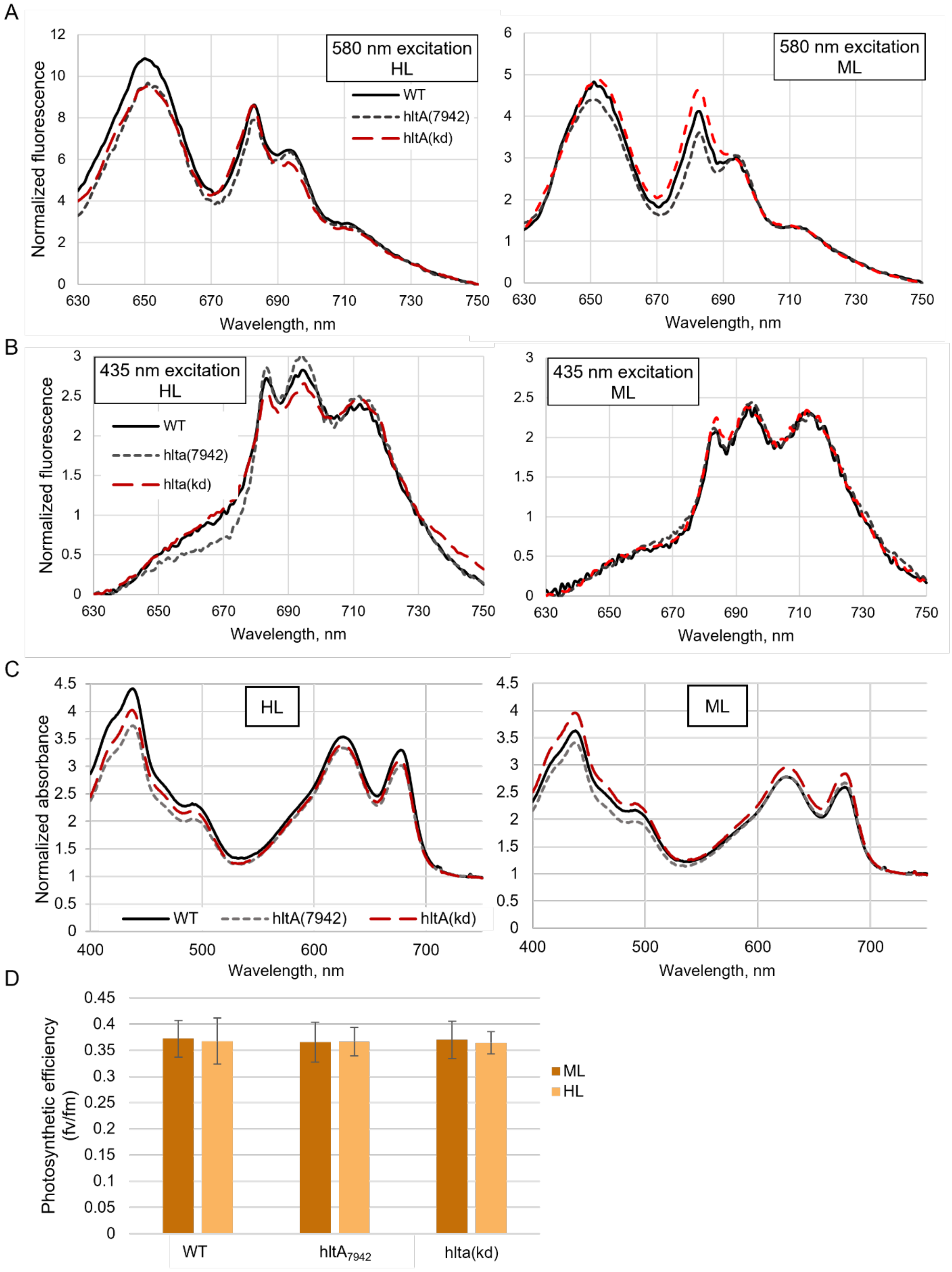

Figure S1. Photosynthetic phenotype and spectra analysis of *hltA* mutant under increasing light. (A-B) Low temperature (77K) fluorescence spectra of WT and *hltA* mutants cultivated under ML_L (500 µmol photons m^{-2} s^{-1}) and HL_L (1500 µmol photons m^{-2} s^{-1}) for 4 hours, normalized to 720 nm. (A) Excitation at 580 nm for PBS, peaks 650-660 nm indicate energy transfer to phycocyanin, 685 nm relates to PSII, peak 695 nm relates to PSI. (B) Excitation of chlorophyll at 435 nm show PSI corresponding to 715 nm, and PSII to 685 nm and 695 nm peaks. (C) Comparison of whole-cell absorbance spectra grown in ML_L or HL_L. To facilitate comparisons, spectra were normalized to 720 nm. Spectra shown are representative data from three biological replicates. (D) Flash induced chlorophyll fluorescence decay kinetics analysis for WT and *hltA* mutants. Bars represent mean Fv/Fm ratio ± standard deviation from three independent replicates.

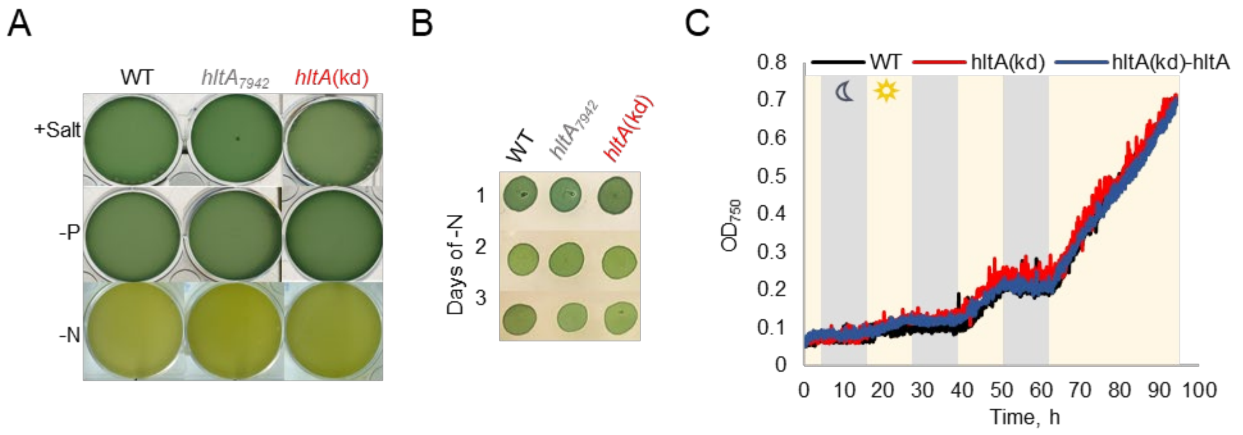

Figure S2. Mutations to HltA do not affect general stress growth or recovery. Non-light environmental stress analysis of *hltA* mutants. (A) Phenotype of cultures after 4 days of high salt, nitrogen starvation, and phosphorus starvation, grown in 6-well plates. (B) Recovery on BG11 plates after 1-3 days of nitrogen-depletion stress. (C) Growth curves of wild-type and *hltA* strains in 12 hr light/12 hr dark conditions followed by constant light. Light conditions were 75 μmol photons m^{-2} s^{-1}, with bubbling 1% CO_2. (A-B) Experiments were performed at low light with ambient air.

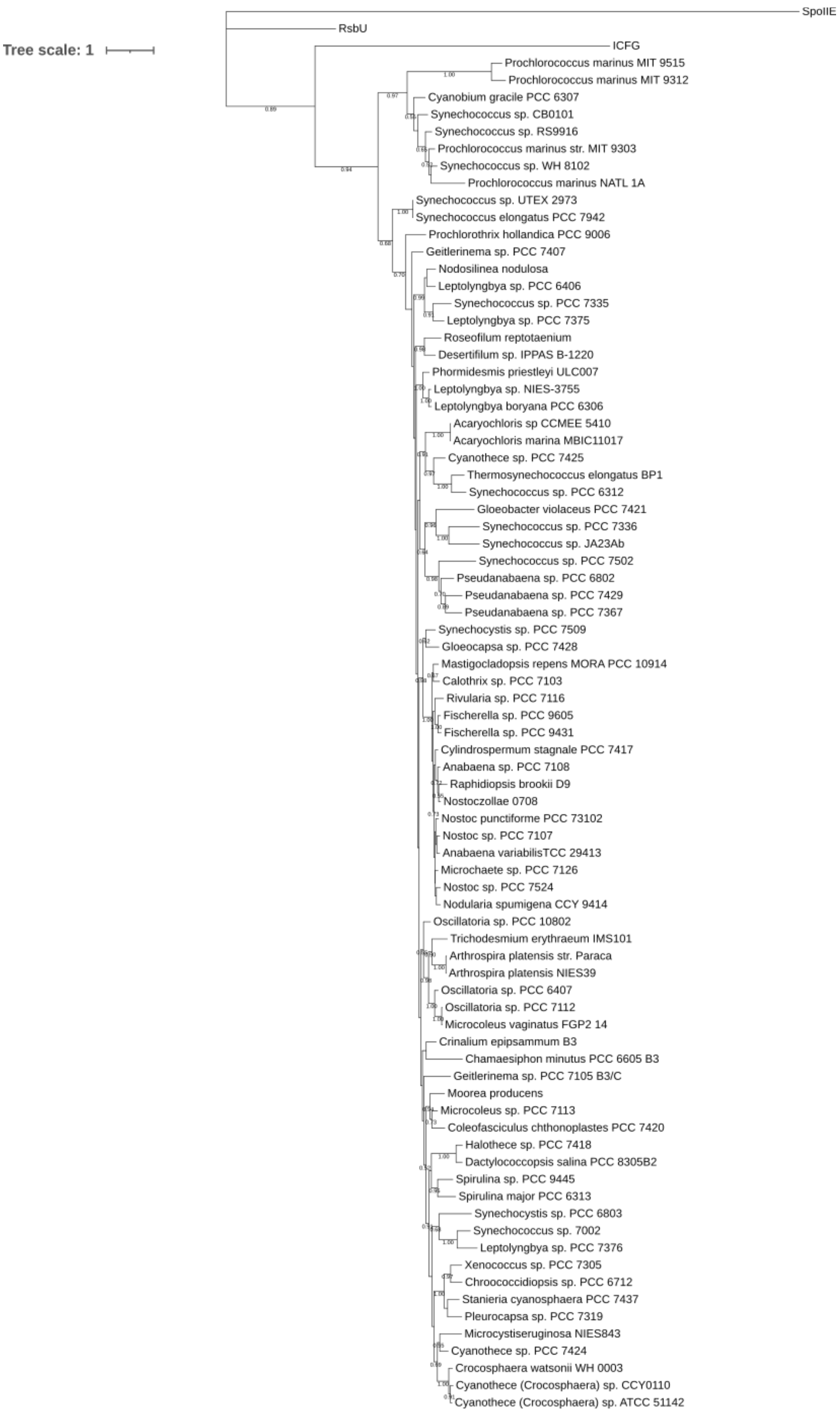

Figure S3. Cyanobacterial tree with bootstrap values (n=1000) and outgroups.

Table S1: Strains used in this study

Nucleotide position	Locus tag	Gene function/ Annotation	Strain Name	Source
2973-WT	—	Control		Yu, Liberton et al. 2015 (1)
pUH24	—	Cure 2973 of plasmid	JU161	Ungerer, 2018 (2)
126938	M744_00705	Hypothetical protein	Ju162	Ungerer, 2018 (2)
236706	M744_1335	ATP synthase F0F1 alpha	Ju129	Ungerer, 2018 (2)
474883	—	Noncoding 101 bp upstream of rpaA	JU212	Ungerer, 2018 (2)
475352	M744_2605	rpaA, SNP I, circadian response regulator	Ju144	Ungerer, 2018 (2)
475390	M744_2605	rpaA, SNP II, circadian response regulator	Ju144	Ungerer, 2018 (2)
610804	M744_3335	Manganese ABC transporter ATP-binding	JU177	Ungerer, 2018 (2)
705129	M744_03855	Guanylate cyclase/ Serine phosphatase/ HltA	hltA$_{7942}$	Ungerer, 2018 (2)
891346	M744_4780	PpnK – NAD+ kinase	JU175	Ungerer, 2018 (2)
1042862	—	Noncoding, 362 bp upstream of *ycfA*	JU214	Ungerer, 2018 (2)
1080351	M744_5865	Hypothetical protein	Ju165	Ungerer, 2018 (2)
1113358	M744_6025	Molecular chaperone DnaK	Ju145	Ungerer, 2018 (2)
1222741	M744_6570	Hydrolase	Ju146	Ungerer, 2018 (2)
1237531	M744_6650	CTP synthetase	Ju179	Ungerer, 2018 (2)
1238113	M744_6650	CTP synthetase	Ju181	Ungerer, 2018 (2)
1273424	M744_6850	Chorismate mutase	JU155	Ungerer, 2018 (2)
1533430	—	Noncoding, 120 bp upstream of COP23	JU216	Ungerer, 2018 (2)
1619501	M744_8615	DNA-directed RNA polymerase β subunit	JU127	Ungerer, 2018 (2)
1718274	M744_11685	Anthranilate synthase	JU154	Ungerer, 2018 (2)
1741647	—	Noncoding, downstream of two genes	JU218	Ungerer, 2018 (2)
2139224	—	Noncoding, 130 bp upstream of histone like protein	JU220	Ungerer, 2018 (2)

2249045	M744_12130	Long-chain fatty acid CoA ligase	JU192	Ungerer, 2018 (2)
2339502	M744_12130	Long-chain fatty acid CoA ligase	JU153	Ungerer, 2018 (2)
2347222	—	Noncoding, 37 bp upstream of hypothetical	JU208	Ungerer, 2018 (2)
2348957	—	Noncoding, 26 bp upstream of hypothetical	JU210	Ungerer, 2018 (2)
2364720	M744_12285	Glutamate synthase	JU130	Ungerer, 2018 (2)
2608863	M744_13540	Photosystem I assembly protein	JU128	Ungerer, 2018 (2)
Multiple	M744_2605/noncoding	rpaA, SNP I SNPII, noncoding promoter SNP combined	JU221	Ungerer, 2018 (2)
Multiple	M744_1335 and M744_4780	ATP Synthase and ppnk combined	JU186	Ungerer, 2018 (2)
Multiple	M744_4780, M744_1335 and M744_6650	3 genes reverted	JU193	Ungerer, 2018 (2)
Multiple	M744_4780, M744_1335, M744_6850	3 genes reverted	JU198	Ungerer, 2018 (2)
Multiple	M744_4780, M744_1335, M744_6850, and M744_6650	4 genes reverted	Ju196	Ungerer, 2018 (2)
-	M744_03855	M744_03588 knock-down	hltA(kd)	This study
-	M744_03855	M744_03588 complementation	*hltA(kd)-hltA*	This study

Table S2: HltA homologs in non-cyanobacteria

Scientific Name	Metagenome?	Max Score	Total Score	Query Cover	E value	Per. ident	Acc. Len	Accession
Tatlockia sp.	yes	514	514	94%	5.00E-177	60.5	458	MBA2750123.1
Chloroflexaceae bacterium	yes	493	493	93%	1.00E-168	57.08	474	NJL83976.1
Propionibacteriaceae bacterium	yes	428	428	93%	4.00E-143	50.68	469	MAR52998.1
Gemmatimonadaceae bacterium	yes	414	414	93%	5.00E-138	52.18	436	MBC8122912.1
Anaerolineae bacterium	yes	411	411	94%	5.00E-137	51.26	428	MBC7881039.1
Acidobacteriaceae bacterium	yes	212	212	93%	2.00E-56	33.48	879	NUQ27114.1
Acidobacteria bacterium	yes	208	208	93%	4.00E-55	33.04	879	MBW8747969.1
Granulicella sp. S190	-	207	207	91%	7.00E-55	34.38	874	WP_158945906.1
Terriglobus albidus	-	206	206	93%	2.00E-54	32.6	879	WP_147650223.1
Acidobacteria bacterium	yes	202	202	92%	4.00E-54	31.47	628	MBV9957074.1
Acidobacteria bacterium 13_1_40CM_4_58_4	yes	197	197	90%	6.00E-54	33.41	427	OLC97355.1
Bryobacterales bacterium	yes	204	204	92%	8.00E-54	33.48	862	MBL8228477.1
Acidobacteria bacterium	yes	198	198	92%	5.00E-53	31.92	581	PYS47485.1
Edaphobacter lichenicola	-	196	196	91%	8.00E-51	32.74	824	WP_179636654.1
Chloroflexi bacterium	yes	193	193	92%	9.00E-51	32.65	656	MBM3135102.1
Edaphobacter lichenicola	-	196	196	91%	1.00E-50	32.59	874	MBB5329045.1
Edaphobacter lichenicola	-	195	195	91%	2.00E-50	30.49	876	WP_183789934.1
Edaphobacter lichenicola	-	195	195	91%	2.00E-50	33.63	876	WP_179581468.1
Edaphobacter modestus	-	195	195	94%	2.00E-50	31.42	874	WP_130418875.1
Edaphobacter lichenicola	-	194	194	91%	3.00E-50	32.88	882	WP_183812128.1
Acidobacteriia bacterium	yes	194	194	92%	5.00E-50	32.68	869	MBZ5535594.1
Acidobacteriia bacterium	yes	193	193	94%	8.00E-50	31.6	877	MBZ5640185.1
Candidatus Poribacteria bacterium	yes	186	186	93%	1.00E-49	30.09	450	RKY01516.1
Edaphobacter lichenicola	-	192	192	91%	3.00E-49	32.81	877	WP_183767659.1
Edaphobacter aggregans	-	191	191	91%	7.00E-49	31.77	877	WP_051978591.1

Acidobacteriia bacterium	yes	190	190	92%	1.00E-48	32.28	850	MBZ5724199.1
Edaphobacter sp. 4G125	yes	189	189	91%	2.00E-48	32.09	879	WP_186695529.1
Vicinamibacteria bacterium	yes	188	188	92%	6.00E-48	32.09	870	MBK5255026.1
Edaphobacter aggregans	-	187	187	91%	1.00E-47	30.79	865	WP_125486341.1
Acidobacteriaceae bacterium	yes	186	186	92%	1.00E-47	32.23	781	MBV8895472.1
Chloroflexi bacterium	yes	180	180	89%	1.00E-47	32.03	421	MBU0492686.1
Geobacter argillaceus	yes	186	186	85%	1.00E-47	30.75	727	TWJ14039.1

Table S3: Primer sequences used in this study

**Deletion**	
Frag1_R_KanR	gctgcccgtcctctttgaacttttgctttgccacggaac
Frag1_F_KanR	cgatccatcgtctcgttagaaaaactcatcgagcatcaaatgaaactgc
Frag_2_F_HltA_DS	gatgagtttttctaacgagacgatggatcggtg
Frag2_R_HltA_DS	ctgtgactggtgagttgcgttcaaagccgagtaatgc
Frag3_R_PUC	tcggctttgaacgcaactcaccagtcacagaaaagcat
Frag3_F_PUC	cctcgattgtctcaagatcctgcctcgcgcg
Frag4_F_HltA_US	gcgcgaggcaggatcttgagacaatcgaggcactgttgctac
Frag4_R_HltA_US	caaagcaaaagttcaaagaggacgggcagct
**Complementation**	
Gm_Rev_Comp	caccgatccatcgtctcgctcgaattgacataagcctgttcgg
SL3360_FOR	cgggccgtttgaaccaggcatcaaataaaacgaaag
HltA_UP_Comp	gtcgtcataagaggacgggcaagaggacgggcagct
HltA_DS_Comp	caggcttatgtcaattcgagcgagacgatggatcggtgag
HltA_for_Comp	ccgtcctcttatgacgacgctcccctattt
HltA_Rev_p2103	tttgatgcctggttcaaacggcccgcagg
**Sequencing**	
Chr_HltA_UP_seq	taaccgttgggaacttttctcc
Chr_HltA_DS_seq	tctacagcagatcgctcacc

Table S4: Orthologs in the cyanobacterial core from cyanobacterial genomes.

Genome Name	JGI ID	50%	40%	Locus Tag
Acaryochloris marina MBIC11017	641228474	1		
Acaryochloris sp CCMEE 5410	2513237397	1		
Acidobacteria bacterium Mor1	2630968265	1		
Anabaena cylindrica PCC 7122 NIES-19	2802429329	1		
Anabaena sp. PCC 7108	2506485002	1		
Anabaena sp. WA102	2651869734	1		
Anabaena variabilis ATCC 29413	646564504	1		
Anabaenopsis circularis NIES-21	2775506824	1		
Aphanizomenon flos-aquae 2012/KM1/D3	2630968589	1		
Aphanocapsa montana BDHKU210001	2627853929	1		
Aphanothece cf. minutissima CCALA 015	2786546929	1		
Aphanothece hegewaldii CCALA 016	2786546931	1		
Arthrospira platensis C1	2507262036	1		
Arthrospira platensis NIES-39	650377906	1		
Arthrospira platensis Paraca	645951858	1		
Arthrospira platensis YZ	2687453185	1		
Arthrospira sp. TJSD091	2645728008	1		
Aulosira laxa NIES-50	2775506816	1		
Baaleninema simplex PCC 7105	2510065011	1		
Calothrix brevissima NIES-22	2775506883	1		
Calothrix desertica PCC 7102	2509887024	1		
Calothrix elsteri CCALA 953	2791355003	1		
Calothrix parasitica NIES-267	2775506882	1		
Calothrix sp. NIES-2098	2775506880	1		
Calothrix sp. NIES-2100	2775506876	1		
Calothrix sp. NIES-3974	2775506881	1		
Calothrix sp. NIES-4101	2775506878	1		
Calothrix sp. NIES-4105	2775506879	1		
Calothrix sp. PCC 7103	2507262048	1		
Candidatus Atelocyanobacterium thalassa ALOHA	646311970	1		
Candidatus Atelocyanobacterium thalassa SIO64986	2617271233	1		
Chamaesiphon minutus PCC 6605	2510436000	1		
Chamaesiphon polymorphus CCALA 037	2788500265	1		
Chlorogloea sp. CCALA 695	2788500227	1		
Chlorogloeopsis fritschii PCC 6912	2512047082	1		

Chlorogloeopsis fritschii PCC 9212	2548877023	1		
Chondrocystis sp. NIES-4102	2775506821	1		
Chroococcales cyanobacterium CENA595	2651869645	1		
Chroococcidiopsis sp. PCC 6712	2505679029	1		
Chroococcidiopsis sp. TS-821	2831353354	1		
Chrysosporum ovalisporum UAM-MAO	2713896943	1		
Coleofasciculus chthonoplastes PCC 7420	647533184	1		
Crinalium epipsammum PCC 9333	2504643013	1		
Crocosphaera chwakensis CCY 0110	640612201	1		
Crocosphaera subtropica BH68	641522622	1		
Crocosphaera watsonii WH 0003	2531839001	1		
Crocosphaera watsonii WH 0005	2636415574	1		
Crocosphaera watsonii WH 0401	2503283007	1		
Crocosphaera watsonii WH 0402	2627853717	1		
Crocosphaera watsonii WH 8501	2503283009	1		
Cuspidothrix issatschenkoi CHARLIE-1	2788499917	1		
Cyanobacterium aponinum PCC 10605	2503707009	1		
cyanobacterium PCC 7702	2512564012	1		
Cyanobium gracile PCC 6307	2508501011	1		
Cyanobium sp. NIES-981	2757320922	1		
Cyanosarcina cf. burmensis CCALA 770	2788500226	1		
Cyanothece sp. PCC 7425	643348534	1		
Cylindrospermopsis raciborskii CENA302	2831626777	1		
Cylindrospermopsis raciborskii CS-505	647000233	1		
Cylindrospermopsis raciborskii S07	2831630099	1		
Cylindrospermopsis raciborskii S14	2831783282	1		
Cylindrospermum sp. NIES-4074	2837197950	1		
Cylindrospermum stagnale PCC 7417	2509601025	1		
Dactylococcopsis salina PCC 8305	2509276056	1		
Elainella saxicola E1	2786546530	1		
Euhalothece sp. PCC 7418	2786546930	1		
Fischerella muscicola CCMEE 5323	2806311008	1		
Fischerella muscicola PCC 7414	2548876996	1		
Fischerella muscicola SAG 1427-1	2548876995	1		
Fischerella sp. NIES-3754	2687453106	1		
Fischerella sp. NIES-4106	2775506903	1		
Fischerella sp. PCC 9339	2516653082	1		
Fischerella sp. PCC 9431	2512875027	1		

Fischerella sp. PCC 9605	2516143000	1		
Fischerella thermalis CCMEE 5196	2802429611	1		
Fischerella thermalis CCMEE 5198	2802429574	1		
Fischerella thermalis CCMEE 5201	2802429601	1		
Fischerella thermalis CCMEE 5205	2802429602	1		
Fischerella thermalis CCMEE 5208	2802429592	1		
Fischerella thermalis CCMEE 5268	2802429570	1		
Fischerella thermalis CCMEE 5273	2802429572	1		
Fischerella thermalis CCMEE 5318	2802429576	1		
Fischerella thermalis CCMEE 5319	2802429575	1		
Fischerella thermalis CCMEE 5328	2802429612	1		
Fischerella thermalis CCMEE 5330	2802429593	1		
Fischerella thermalis PCC 7521	2548876998	1		
Fischerella thermalis WC119	2802429584	1		
Fischerella thermalis WC245	2806310400	1		
Fischerella thermalis WC341	2802429581	1		
Fischerella thermalis WC441	2802429594	1		
Fischerella thermalis WC527	2802429598	1		
Fischerella thermalis WC538	2802429597	1		
Fischerella thermalis WC542	2802429596	1		
Fischerella thermalis WC558	2802429588	1		
Fischerella thermalis WC559	2802429589	1		
Fortiea contorta PCC 7126	2509601027	1		
Geitlerinema sp. PCC 7407	2503538020	1		
Geitlerinema sp. PCC 9228 re-assembly	2660238729	1		
Geminocystis herdmanii PCC 6308	2509601046	1		
Geminocystis sp. NIES-3708	2675903523	1		
Geminocystis sp. NIES-3709	2671180423	1		
Gloeobacter kilaueensis JS1	2558309063	1		
Gloeobacter violaceus PCC 7421	637000121	1		
Gloeocapsa sp. PCC 7428	2503754017	1		
Gloeothece citriformis PCC 7424	643348533	1		
Halomicronema hongdechloris C2206	2757320732	1		
Halothece sp. PCC 7418	2503538028	1		
Hassallia byssoidea VB512170	2627853647	1		
Kamptonema formosum PCC 6407	2508501075	1		
Kamptonema sp. PCC 6506	648276706	1		
Leptolyngbya boryana IAM M-101	2773857838	1		
Leptolyngbya boryana PCC 6306	2509601031	1		
Leptolyngbya sp. Heron Island J	2576861623	1		
Leptolyngbya sp. JSC-1	2022827000	1		
Leptolyngbya sp. NIES-2104	2690315883	1		

Leptolyngbya sp. NIES-3755	2687453458	1		
Leptolyngbya sp. O-77	2681813363	1		
Leptolyngbya sp. PCC 6406	2517572073	1		
Leptolyngbya sp. PCC 7375	2509601039	1		
Leptolyngbya sp. PCC 7376	2503754048	1		
Limnospira maxima CS-328	642979357	1		
Lyngbya aestuarii BL J	2576861326	1		
Lyngbya sp. PCC 8106	639857035	1		
Mastigocladopsis repens PCC 10914	2517093042	1		
Mastigocladus laminosus UU774	2630968419	1		
Merismopedia glauca CCAP 1448/3	2802429465	1		
Microcoleus sp. PCC 7113	2509276031	1		
Microcoleus vaginatus FGP-2	2506210028	1		
Microcoleus vaginatus PCC 9802	2505119011	1		
Microcystis aeruginosa CHAOHU 1326	2831959033	1		
Microcystis aeruginosa FACHB-524	2831587555	1		
Microcystis aeruginosa NIES-2481	2721755494	1		
Microcystis aeruginosa NIES-2549	2645727547	1		
Microcystis aeruginosa NIES-298	2831434989	1		
Microcystis aeruginosa NIES-843	641522640	1		
Microcystis aeruginosa PCC 7005	2585427674	1		
Microcystis aeruginosa PCC 7806	2510917005	1		
Microcystis aeruginosa PCC 7806SL	2751185885	1		
Microcystis aeruginosa PCC 9717	2534681680	1		
Microcystis aeruginosa PCC 9806	2534681685	1		
Microcystis aeruginosa PCC 9807	2534681684	1		
Microcystis aeruginosa TAIHU98	2541047129	1		
Microcystis panniformis FACHB-1757	2645727631	1		
Microcystis sp. 0824	2831619920	1		
Microcystis sp. MC19	2791355144	1		
Microcystis sp. T1-4	2531839541	1		
Moorea bouillonii PNG5-198	2515154003	1		
Moorea producens PAL 15AUG08-1	2630968268	1		
Nodosilinea nodulosa PCC 7104	2509601026	1		
Nodularia sp. NIES-3585	2775506862	1		
Nodularia spumigena CCY9414	2562617131	1		
Nostoc carneum NIES-2107	2775506992	1		
Nostoc commune NIES-4072	2831442766	1		
Nostoc flagelliforme CCNUN1	2775506989	1		
Nostoc linckia NIES-25	2833620646	1		
Nostoc linckia z15	2843045665	1		
Nostoc linckia z2	2845869039	1		

Nostoc linckia z4	2845887725	1		
Nostoc linckia z7	2845860650	1		
Nostoc linckia z8	2845911424	1		
Nostoc linckia z9	2845847289	1		
Nostoc punctiforme PCC 73102	642555144	1		
Nostoc sp. 210A	2837700758	1		
Nostoc sp. ATCC 53789	651717004	1		
Nostoc sp. CENA543	2788500609	1		
Nostoc sp. NIES-2111	2775506991	1		
Nostoc sp. NIES-3756	2671180707	1		
Nostoc sp. NIES-4103	2775506988	1		
Nostoc sp. PCC 7107	2503707008	1		
Nostoc sp. PCC 7120	637000199	1		
Nostoc sp. PCC 7524	2509601032	1		
Nostoc sphaeroides Kutzing En	2841035561	1		
Nostocales cyanobacterium HT-58-2	2758568003	1		
Okeania hirsuta PAB10Feb10-1	2831758409	1		
Oscillatoria nigro-viridis PCC 7112	2503982035	1		
Oscillatoria sp. 08	2721755987	1		
Oscillatoria sp. PCC 10802	2509276047	1		
Phormidesmis priestleyi ULC007	2802429464	1		
Phormidium willei BDU 130791	2744054668	1		
Planktothrix agardhii NIES-204	2833584432	1		
Planktothrix agardhii NIVA-CYA 15	2506381000	1		
Planktothrix agardhii NIVA-CYA 56/3	2523533592	1		
Planktothrix NIVA-CYA405	2505679099	1		
Planktothrix paucivesiculata PCC 9631	2791355021	1		
Planktothrix prolifica NIVA-CYA 406	2602041638	1		
Planktothrix prolifica NIVA-CYA 540	2512875029	1		
Planktothrix prolifica NIVA-CYA 98	2506381013	1		
Planktothrix rubescens NIVA-CYA 407	2602041655	1		
Planktothrix serta PCC 8927	2791355023	1		
Planktothrix sp. 585	2506381002	1		
Planktothrix sp. PCC 11201	2831592450	1		
Planktothrix tepida PCC 9214	2791355022	1		
Pleurocapsa minor PCC 7327	2509276061	1		
Pleurocapsa sp. CCALA 161	2788500296	1		
Pleurocapsa sp. PCC 7319	2509601013	1		
Prochlorococcus marinus MIT9211	641228501	1		
Prochlorococcus marinus MIT9303	640069323	0	1	

Prochlorococcus marinus MIT9312	637000210	0	1	
Prochlorococcus marinus MIT9515	640069324	0	1	
Prochlorococcus marinus NATL1A	640069325	0	1	
Prochlorococcus marinus NATL2A	637000212	0	1	
Prochloron didemni P1-Palau	2510461037	1		
Prochloron didemni P3-Solomon	2600255115	1		
Prochloron didemni P4-Palau	2510461040	1		
Prochloron didemni P4-Papua_New_Guinea	2600255113	1		
Prochlorothrix hollandica PCC 9006	2509276045	1		
Pseudanabaena biceps PCC 7429	2504557005	1		
Pseudanabaena sp. PCC 6802	2506783054	1		
Pseudanabaena sp. PCC 7367	2504643012	1		
Raphidiopsis brookii D9	647000303	1		
Richelia intracellularis HM01	2600255025	1		
Rivularia sp. PCC 7116	2510065008	1		
Scytonema hofmanni PCC 7110	2551306141	1		
Scytonema hofmanni UTEX 2349	2507262016	1		
Scytonema sp. NIES-2130	2802429304	1		
Sphaerospermopsis aphanizomenoides BCCUSP55	2728369585	1		
Sphaerospermopsis kisseleviana NIES-73	2775506855	1		
Spirulina major PCC 6313	2506520014	1		
Spirulina subsalsa PCC 9445	2506520011	1		
Stanieria cyanosphaera PCC 7437	2503754019	1		
Stanieria sp. NIES-3757	2775506853	1		
Stenomitos frigidus ULC18	2788500495	1		
Synechococcus elongatus PCC 6301	637000307	1		
Synechococcus elongatus PCC 7942	637000308	1		
Synechococcus lividus PCC 6715	2775507028	1		
Synechococcus sp. 1G10	2831559763	1		
Synechococcus sp. 7002	2708742468	1		
Synechococcus sp. 8F6	2834131510	1		
Synechococcus sp. BO 8801	2838880839	1		
Synechococcus sp. CB0101 GCA_000179235.1	649990022	1		
Synechococcus sp. CC9605	637000310	1		
Synechococcus sp. GFB01	2636415834	1		
Synechococcus sp. JA-2-3B'a(2-13)	637000312	1		
Synechococcus sp. JA-3-3Ab	637000313	0	1	
Synechococcus sp. KORDI-100	2507262013	1		
Synechococcus sp. KORDI-49	2507262011	1		
Synechococcus sp. MIT9504	2823440501	0	1	
Synechococcus sp. MIT9508	2823447967	0	1	

Synechococcus sp. OG1	2708742537	1		
Synechococcus sp. PCC 6312	2509276030	1		
Synechococcus sp. PCC 7003	2504643000	1		
Synechococcus sp. PCC 7117	2504643001	1		
Synechococcus sp. PCC 73109	2504643003	1		
Synechococcus sp. PCC 7335	647533236	1		
Synechococcus sp. PCC 7336	2506520048	1		
Synechococcus sp. PCC 7502	2508501041	1		
Synechococcus sp. PCC 8807	2504643002	1		
Synechococcus sp. RS9916	639857007	1		
Synechococcus sp. RS9917	638341213	1		
Synechococcus sp. SynAce01	2718218441	1		
Synechococcus sp. UTEX 2973	2630968581	1		
Synechococcus sp. WH 8016	2507262052	0	1	
Synechococcus sp. WH 8101	2837243552	1		
Synechococcus sp. WH 8103	2687453380	1		
Synechococcus sp. WH8102	637000314	0	1	
Synechocystis sp. GT-S, PCC 6803	651053076	1		
Synechocystis sp. IPPAS B-1465	2834893250	1		
Synechocystis sp. PCC 6803	2561511183	1		
Synechocystis sp. PCC 6803 GT-I	2513237196	1		
Synechocystis sp. PCC 6803 PCC-N	2513237195	1		
Synechocystis sp. PCC 6803 PCC-P	2524023216	1		
Synechocystis sp. PCC 6803 PCC6803	2687453425	1		
Synechocystis sp. PCC 7509	2517572074	1		
Thermosynechococcus elongatus BP-1	637000320	1		
Thermosynechococcus elongatus PKUAC-SCTE542	2833578469	1		
Thermosynechococcus sp. NK55a	2597489959	1		
Thermosynechococcus vulcanus NIES-2134	2837246461	1		
Tolypothrix campylonemoides VB511288	2636415724	1		
Tolypothrix sp. NIES-4075	2775506872	1		
Tolypothrix sp. PCC 7601	2648501203	1		
Trichodesmium erythraeum IMS101	2627853943	1		
Trichodesmium thiebautii H9-4	2627854162	1		
Trichormus azollae 0708	648028001	1		
Vulcanococcus limneticus LL	2831889438	1		
Xenococcus sp. PCC 7305	2508501034	1		
Cyanobacterium sp. UCYN-A2	2528768021	1		
Leptolyngbya sp. FACHB-17	2914460412	1		Ga0477554_77_57784_59223
Lyngbya confervoides BDU141951	2636415444	1		Ga0078340_1274967

Arthrospira sp. TJSD092	2788500392	1		Ga0302594_112518
Halomicronema excentricum Lakshadweep	2881457148	1		Ga0440342_08_1332538_1333914
Desertifilum sp. FACHB-866	2914193409	1		Ga0477590_38_163406_164860
Hormoscilla sp. GM102CHS1	2767802581	1		Ga0248556_118618
Trichocoleus sp. FACHB-591	2909842179	1		Ga0477545_097_53865_55289
Planktothricoides sp. FACHB-1370	2909811262	1		Ga0477621_061_12821_14314
Calothrix sp. 336/3	2654587977	1		Ga0111347_112300
Nodosilinea sp. LEGE 07088	2914093931	1		Ga0481340_374_294_1745
Planktothrix agardhii NIVA-CYA 34	2506381012	1		DRAFT34_00006200
Aphanothece sacrum FPU1	2882194956	1		Ga0441946_03_106947_108311
Leptolyngbya boryana dg5	2773857877	1		Ga0226171_122155
Microcoleus sp. FACHB-53	2909751754	1		Ga0477548_40_735077_736534
Microcoleus sp. FACHB-45	2914448719	1		Ga0477549_103_17610_19097
Alkalinema sp. FACHB-956	2909475975	1		Ga0477592_01_238294_239772
Nostoc sp. Moss5	2630968270	1		Ga0080674_1044266
Oscillatoria sp. FACHB-1406	2909541658	1		Ga0477622_38_124289_125779
Pseudanabaena sp. FACHB-2040	2910123500	1		Ga0477579_05_45452_46903
Limnospira fusiformis SAG 85.79	2883428434	1		Ga0439576_01_2573409_2574797
Trichocoleus sp. FACHB-6	2910177317	1		Ga0478396_023_20090_21568
Hormoscilla spongeliae SP12CHS1	2643221529	1		Ga0102016_12737
Nodosilinea sp. FACHB-13	2914240099	1		Ga0477557_16_205502_206956
Microcoleus sp. FACHB-DQ6	2909511626	1		Ga0477525_304_35962_37449
Trichocoleus sp. FACHB-832	2909734344	1		Ga0478390_031_190248_191714
Tychonema sp. LEGE 07199	2917535239	1		Ga0481343_018_20946_22433
Aphanothece sacrum FPU3	2882199549	1		Ga0441947_90_18053_19417
Leptolyngbya sp. FACHB-16	2909884012	1		Ga0478399_108_30026_31450
Desertifilum sp. FACHB-1129	2909863786	1		Ga0477589_12_162802_164256
Nodosilinea sp. LEGE 06152	2914139253	1		Ga0481336_23_72231_73604
Microcoleus sp. FACHB-68	2909683005	1		Ga0477541_15_8875_10440
Limnospira indica PCC 8005	2751185742	1		Ga0175806_112502
Leptolyngbya sp. FACHB-671	2917594760	1		Ga0478394_065_173224_174663
Planktothricoides sp. SR001	2636416084	1		Ga0099329_102724
Phormidium sp. FACHB-592	2910170622	1		Ga0477544_086_224702_226219
Brasilonema bromeliae SPC951	2887007579	1		Ga0442301_024_24027_25430
Nodosilinea sp. FACHB-131	2914543266	1		Ga0477533_44_19941_21395
Oscillatoria sp. 01	2721755986	1		Ga0182244_10085
Anabaena subtropica FACHB-260	2914484530	1		Ga0477595_022_2391_3782
Oscillatoria sp. FACHB-1407	2909702043	1		Ga0477613_12_249081_250523
Gloeocapsopsis sp. AAB1	2886601917	1		Ga0452604_046_82836_84248
Oscillatoriales cyanobacterium JSC-12	2510065010	1		OsccyDRAFT_2380
Microcoleus sp. FACHB-672	2919943560	1		Ga0477542_13_214476_216281
Arthrospira platensis FACHB-439	2910111508	1		Ga0477645_254_34109_35569

Cyanobacteria bacterium SB-MAG 10	2806310582	1		Ga0316330_105448
Synechococcus elongatus FACHB-805	2909627430	1		Ga0477657_01_98884_100272
Phormidesmis sp. BC1401	2627853604	1		Ga0079976_101773
Nodosilinea sp. P-1105	2887192876	1		Ga0442552_122_33139_34593
Microcoleus vaginatus HSN003	2721756094	1		Ga0181879_3348
Gloeocapsopsis crepidinum LEGE 06123	2914404550	1		Ga0481332_142_3213_4625
Microcoleus sp. FACHB-84	2914530306	1		Ga0477537_011_17610_19097
Chroococcidiopsis cubana CCALA 043	2802429427	1		Ga0303535_17112
Trichocoleus sp. FACHB-69	2919978586	1		Ga0478393_014_44108_45574
Desertifilum sp. FACHB-868	2919917403	1		Ga0477591_44_163422_164876
Trichocoleus sp. FACHB-90	2909429755	1		Ga0478389_071_44256_45734
Coleofasciculus sp. FACHB-SPT9	2913975864	1		Ga0478388_06_223142_224560
Cyanobacteria bacterium SB-MAG 34	2806310597	1		Ga0316354_10324
Leptolyngbya boryana NIES-2135	2811995071	1		Ga0263400_132157
Leptolyngbya sp. KIOST-1	2617271295	1		Ga0062155_102718
Trichormus variabilis FACHB-319	2910117387	1		Ga0477597_40_21191_22582
Coleofasciculus sp. FACHB-129	2917565766	1		Ga0477535_43_80789_82207
Arthrospira platensis NIES-46	2886438073	1		Ga0443361_091_4708_6186
Trichocoleus sp. FACHB-40	2909600524	1		Ga0477550_042_43251_44717
Microcoleus sp. FACHB-61	2914012002	1		Ga0478387_42_52856_54343
Microcoleus vaginatus PCC9802	2737471644	1		Ga0181881_3667
Phormidium sp. FACHB-77	2920022140	1		Ga0477540_50_148241_149632
Tychonema sp. LEGE 07203	2914114882	1		Ga0481344_083_87815_89302
Romeria aff. gracilis LEGE 07310	2917549083	1		Ga0481347_118_41779_43245
Microcoleus sp. FACHB-831	2909389138	1		Ga0477538_35_29329_30762
Nostoc cycadae WK-1	2788500613	1		Ga0308443_100337
Microcoleus sp. LEGE 07076	2914058381	1		Ga0481338_155_12622_14112
Planktothrix sp. FACHB-1365	2909947601	1		Ga0477615_24_20584_22056
Chroococcidiopsis cubana SAG 39.79	2887013700	1		Ga0442425_180_3376_4773
Oscillatoria acuminata PCC 6304	2509276028	1		Oscil6304_4750
Leptolyngbya sp. BC1307	2883213363	1		Ga0442178_003_99560_101098
Chroococcidiopsis thermalis PCC 7203	2503538021	1		Chro_2322
Leptolyngbya sp. FACHB-711	2909823187	1		Ga0478392_270_2587_4032
Coleofasciculus sp. LEGE 07081	2913947054	1		Ga0481339_215_81462_82937
Leptolyngbya sp. FACHB-238	2919936540	1		Ga0477602_67_35246_36670
Rippkaea orientalis PCC 8801	643348535	1		PCC8801_3662
Planktothricoides sp. FACHB-1261	2909929793	1		Ga0477641_075_12833_14326
Crocosphaera subtropica BH63E	2507262054	1		Cy51472_1291

Neosynechococcus sphagnicola CAUP A 1101	2636415546	1		Ga0072884_1026123
Leptolyngbya sp. FACHB-161	2919991004	1		Ga0477599_56_35252_36676
Nostoc sp. FACHB-145	2909489378	1		Ga0478401_001_174067_175458
Pseudanabaenaceae cyanobacterium LEGE 13415	2914368804	1		Ga0481361_0161_7067_8497
Scytonema tolypothrichoides VB-61278	2645727633	1		Ga0077267_1008273
Leptolyngbya sp. FACHB-402	2909524766	1		Ga0477600_67_35246_36670
Nostoc sp. FACHB-152	2913924747	1		Ga0477610_079_28101_29492
Oculatella sp. LEGE 06141	2914436621	1		Ga0481333_114_105401_106831
Trichocoleus sp. FACHB-262	2909908154	1		Ga0477552_097_65505_66929
Trichocoleus sp. FACHB-46	2920004584	1		Ga0477531_78_48317_49741
Planktothrix mougeotii NIVA-CYA 405	2600254967	1		Ga0052983_04592
Trichormus variabilis 0441	2883181452	1		Ga0443053_01_1648893_1650284
Leptolyngbya sp. 14	2721755988	1		Ga0182246_10115
Arthrospira platensis FACHB-835	2914274526	1		Ga0477652_309_33965_35452
Nostoc spongiaeforme FACHB-130	2909780346	1		Ga0477637_45_185625_187010
Synechococcus elongatus FACHB-242	2919997581	1		Ga0477634_01_848866_850254
Leptolyngbya sp. FACHB-8	2913981770	1		Ga0477539_37_83170_84594
Chroococcidiopsis sp. FACHB-1243	2914549056	1		Ga0477587_054_68436_69833
Phormidium tenue FACHB-1052	2910159883	1		Ga0477578_31_137245_138699
Planktothrix sp. NIVA-CYA407	2523533593	1		cya407DRAFT2_00078
Rippkaea orientalis PCC 8802	644736348	1		Cyan8802_3716
Coleofasciculus sp. FACHB-1120	2909895751	1		Ga0477655_01_216418_217842
Microcoleus sp. IPPAS B-353	2870888317	1		Ga0436823_01_4620671_4622062
Leptolyngbya sp. FACHB-239	2909517787	1		Ga0477601_75_35246_36670
Scytonema millei VB511283	2648501120	1		Ga0077703_10862613
Coleofasciculus sp. FACHB-125	2909665986	1		Ga0477536_26_31979_33397
Brasilonema sp. UFV-L1	2886672058	1		Ga0442300_308_11296_12699
Coleofasciculus sp. FACHB-501	2913990810	1		Ga0477529_36_34280_35698
Microcoleus asticus IPMA8	2909848527	1		Ga0477904_013_76199_77686
Planktothrix sp. st147	2507262029	1		st147_cleanDRAFT_00036470
Microcoleus sp. FACHB-1	2909901336	1		Ga0477558_50_26348_27805
Prochlorococcus sp. MIT9107	2606217692	1		Ga0062505_110337
Coleofasciculus sp. FACHB-542	2913996606	1		Ga0477546_46_92234_93652
Leptolyngbya sp. FACHB-60	2914454751	1		Ga0477543_24_148535_149926
Leptolyngbya sp. FACHB-541	2917587430	1		Ga0477547_128_18615_20054
Geitlerinema sp. FC II	2887311238	1		Ga0443691_0136_12487_13902
Synechococcus leopoliensis UTEX 625a	2517572104	1		synDRAFT_02574
Desertifilum sp. IPPAS B-1220	2751185667	1		Ga0166459_111024
Coleofasciculus sp. FACHB-712	2910092932	1		Ga0478391_028_4493_5911

Dolichospermum sp. FACHB-1091	2909555658	1		Ga0477614_02_72432_73820
Nodosilinea sp. FACHB-141	2909696631	1		Ga0477556_07_146661_148115
Arthrospira platensis FACHB-971	2909621827	1		Ga0477633_254_4697_6184
Phormidium sp. FACHB-1136	2917633114	1		Ga0477607_046_68379_69815
Leptolyngbya sp. FACHB-1624	2909505156	1		Ga0477532_69_38824_40248
Synechococcus elongatus FACHB-1061	2914540435	1		Ga0477656_01_848606_849994
Coleofasciculus sp. FACHB-T130	2909612023	1		Ga0477534_40_27933_29351
Oculatella sp. FACHB-28	2917558857	1		Ga0477551_076_113219_114658
Tolypothrix bouteillei Iicb1	2617271247	1		Ga0060318_1481468
Nodosilinea sp. LEGE 07298	2914023581	1		Ga0481345_249_1023_2477
Microcoleus sp. FACHB-1515	2914207075	1		Ga0477555_32_7010_8449
Coleofasciculus sp. FACHB-64	2914001977	1		Ga0478395_26_109004_110422
Anabaena sp. YBS01	2883409860	1		Ga0439566_01_4661350_4662741

References

1. Yu J, Liberton M, Cliften PF, Head RD, Jacobs JM, Smith RD, Koppenaal DW, Brand JJ, Pakrasi HB. 2015. Synechococcus elongatus UTEX 2973, a fast growing cyanobacterial chassis for biosynthesis using light and CO(2). Sci Rep 5:8132.
2. Ungerer J, Wendt KE, Hendry JI, Maranas CD, Pakrasi HB. 2018. Comparative genomics reveals the molecular determinants of rapid growth of the cyanobacterium Synechococcus elongatus UTEX 2973. Proc Natl Acad Sci U S A doi:10.1073/pnas.1814912115.
